# Comparative Genomics Analyses Reveal Extensive Chromosome Colinearity and Novel Quantitative Trait Loci in *Eucalyptus*

**DOI:** 10.1371/journal.pone.0145144

**Published:** 2015-12-22

**Authors:** Fagen Li, Changpin Zhou, Qijie Weng, Mei Li, Xiaoli Yu, Yong Guo, Yu Wang, Xiaohong Zhang, Siming Gan

**Affiliations:** 1 State Key Laboratory of Tree Genetics and Breeding, Chinese Academy of Forestry, Xiangshan Road, Beijing, 100091, China; 2 Research Institute of Tropical Forestry, Chinese Academy of Forestry, Longdong, Guangzhou, 510520, China; Beijing Forestry University, CHINA

## Abstract

Dense genetic maps, along with quantitative trait loci (QTLs) detected on such maps, are powerful tools for genomics and molecular breeding studies. In the important woody genus *Eucalyptus*, the recent release of *E*. *grandis* genome sequence allows for sequence-based genomic comparison and searching for positional candidate genes within QTL regions. Here, dense genetic maps were constructed for *E*. *urophylla* and *E*. *tereticornis* using genomic simple sequence repeats (SSR), expressed sequence tag (EST) derived SSR, EST-derived cleaved amplified polymorphic sequence (EST-CAPS), and diversity arrays technology (DArT) markers. The *E*. *urophylla* and *E*. *tereticornis* maps comprised 700 and 585 markers across 11 linkage groups, totaling at 1,208.2 and 1,241.4 cM in length, respectively. Extensive synteny and colinearity were observed as compared to three earlier DArT-based eucalypt maps (two maps with *E*. *grandis* × *E*. *urophylla* and one map of *E*. *globulus*) and with the *E*. *grandis* genome sequence. Fifty-three QTLs for growth (10–56 months of age) and wood density (56 months) were identified in 22 discrete regions on both maps, in which only one colocalizaiton was found between growth and wood density. Novel QTLs were revealed as compared with those previously detected on DArT-based maps for similar ages in *Eucalyptus*. Eleven to 585 positional candidate genes were obained for a 56-month-old QTL through aligning QTL confidence interval with the *E*. *grandis* genome. These results will assist in comparative genomics studies, targeted gene characterization, and marker-assisted selection in *Eucalyptus* and the related taxa.

## Introduction

Genetic linkage maps, dense maps in particular, are powerful tools for genomics and molecular breeding studies because they allow for dissection of agronomic traits through map-based localization and isolation of quantitative trait loci (QTLs) or major genes. In plants, high-density maps have already been constructed for a spectrum of crops, such as rice (*Oryza sativa*, 6,591 loci) [1] and maize (*Zea mays*, 20,931 and 14,524 markers with two separate maps) [2]. Moreover, many economically important QTLs have been identified on genetic maps and some have even been cloned to identify the causal genes, e.g. in rice [3–5]. In forest trees, however, dense genetic maps are only recently available with the aid of microarray-based technologies for a few species, such as *Populus trichocarpa* (608 markers) [6], *Picea mariana* (1,111 markers) [7], *Cryptomeria japonica* (1,261 markers) [8], *Pinus taeda* (2,466 markers) [9], *Eucalyptus globulus* (569 and 1,060 markers with two maps) [10], *E*. *grandis*, and *E*. *urophylla* (1,773–2,484 markers) [11–14]. Furthermore, only a few of studies have used dense genetic maps for QTL analysis in forest trees, e.g. *Populus* [15], *Picea* [16], and *Eucalyptus* [17–19].

Fast-growing trees like *Eucalyptus* and *Populus* serve as an important and renewable source of wood for furniture, plywood, fiberboard, pulp, and paper industries as well as biomass for heat, electricity, and bio-fuel production. Quantitative traits such as growth characteristics and wood density are strongly related with yields of wood and biomass and have been the principal targets of breeding efforts. In order to explore the genetic mechanisms underlying such quantitative traits and provide valuable information for breeding practices, QTL mapping has been an attractive approach to investigate the genomic loci of interest [20,21]. Previous studies have identified numerous QTLs for growth and wood density in forest trees [20–23]. Nevertheless, these QTLs are often confined to a single segregating population and must be compared to validate consensus genomic regions of importance among populations within or between species [21]. Whereas map-based positional cloning of causative genes for QTLs remains a challenge in forest trees, the recent availability of several sequenced genomes will facilitate the identification of positional candidate genes by anchoring genetic maps and QTLs onto genome sequences [24], which may represent a key step towards characterization of causative genes. To date, positional candidate genes identified in this way have only been reported in *Populus* [15,24,25] and *Eucalyptus* [17,19].

*Eucalyptus* species and hybrids constitute the most widely planted hardwoods in the world, with global plantations over 20 million hectares [26]. Eucalypts are generally diploid with 2n = 22 chromosomes and have a relatively small genome size (370–700 Mb) [27]. So far, many efforts have been dedicated to develop *Eucalyptus* genomic resources including molecular markers, genetic linkage maps, expressed sequence tags (ESTs), QTLs and whole-genome sequences [22,23,28]. In particular, dense genetic maps have been developed for *E*. *grandis*, *E*. *urophylla* [11–14], and *E*. *globulus* [10], and QTLs for growth and wood density have been localized on several dense maps [17–19]. Moreover, *E*. *grandis* whole genome sequence has been published recently [28]. However, few studies have attempted to compare the genomic regions containing QTLs between different eucalypt species despite of the prevalence of genomic synteny and colinearity within *Eucalyptus* [10,13].

This paper presents dense genetic maps of *E*. *urophylla* and *E*. *tereticornis* with novel QTLs related with growth and wood density. The objectives are 1) to compare the synteny and colinearity with previous diversity arrays technology (DArT) marker based *Eucalyptus* maps and the *E*. *grandis* genome sequence, 2) map QTLs for growth and wood density, and mine the positional candidate genes from the *E*. *grandis* reference genome.

## Materials and Methods

### Mapping Population, Trait Measurement, and Statistical Analyses

The mapping population was derived from a cross between *E*. *urophylla* (Ur, genotype UX-30) and *E*. *tereticornis* (Te, genotype T43-05). A set of 132 full-sibs were used to replace the earlier mapping progeny whose size had been decreased during field maintenance [29]. The full-sibs were clonally propagated for field test.

Cuttings of the mapping cross were planted at Gonghe Town, Guangdong Province (112°51'14'' E, 22°34'24'' N, and around 30 m above sea level) in April 2006. The field trial was laid in a randomized complete block design with six replicates of single tree plots at 2 × 3 m spacing. Tree height (*H*, m) was measured at 10, 23, 32, 44, and 56 months of age (*H*_10_, *H*_23_, *H*_32_, *H*_44_, and *H*_56_, respectively), and diameter at breast height (*D*, cm) was measured at 23, 32, 44, and 56 months (*D*_23_, *D*_32_, *D*_44_, and *D*_56_, respectively). Wood density was indirectly measured at 56 months (*WD*_56_) using Resistograph F-400S (Instrumenta Mechanik Labor GmbH, Wiesloch, Germany) as described [30].

Statistical analyses on trait data were carried out in software SAS/STAT^®^ 8.0 (SAS Institute Inc., Cary, NC, USA). For a given trait, analysis of variance (ANOVA) was performed with Proc MIXED (method REML) to test the significance of variation among genotypes (full-sibs), following a mixed linear model with replicate and genotype (full-sib) as fixed and random effects, respectively. Genotypic and error variance components (V_G_ and V_E_, respectively) were estimated with Proc VARCOMP (method REML) following the same mixed linear model as ANOVA, and clonal repeatability (*H* ^2^) was calculated as *V*_*G*_/(*V*_*G*_ + *V*_*E*_ / 6). Phenotypic correlation coefficients between traits were determined with Proc CORR.

### Ethics Statement

No specific permissions were required for the field location/activities because Research Institute of Tropical Forestry, Chinese Academy of Forestry establishes and manages tree plantations at several locations including the one used in the present study at Gonghe Town, Guangdong Province. These plantations are dedicated to research activities, where field trials were performed strictly following the guidelines formulated by Research Institute of Tropical Forestry, Chinese Academy of Forestry. The field trial did not involve endangered or protected species.

### Molecular Marker Assays

Three molecular marker assays were performed across the mapping population, namely, simple sequence repeats [SSR, including genomic SSR (gSSR) and EST-SSR], EST-derived cleaved amplified polymorphic sequence (EST-CAPS), and DArT. A subtotal of 839 SSRs were tested with the two mapping parents for polymerase chain reaction (PCR) amplification, including 465 EST-SSRs [31–33], 300 gSSRs named with the prefix EMBRA [34], 12 gSSRs with the prefix EMCRC [35], and seven gSSRs with the prefix EUCgSSR [36] as well as 8, 8, 26, and 13 gSSRs with prefixes En, Es, Eg, and El, respectively ([37–39],http://www.ffp.csiro.au/tigr/molecular/eucmsps.html). The SSR genotyping was conducted as reported earlier [40], with MgCl_2_ concentration and melting temperature (*T*_m_) depending on marker. SSRs polymorphic between the parents were then used for genotyping across the mapping population. A set of 91 EST-CAPS markers reported earlier [29] were also included. The DArT array composed of 7,680 informative features was the same as reported earlier [10,12,13]. Low-quality DArT markers were removed based on filtering parameters of ≥ 90.0% reproducibility, ≥ 75.0% call rate, and ≥ 70.0% marker quality *Q* value. The DArT sequences were as deposited in GenBank (accessions HR865291–HR872186, http://www.ncbi.nlm.nih.gov/nucgss) [12].

### Genetic Map Construction and QTL Analysis

Genetic maps were constructed for each of the parents using the regression approach for cross-pollinated population in software JoinMap 4.1 [41]. Marker segregation was tested with a chi-square test, and the distorted markers were included in further mapping given that segregation distortion had little impact on map order or length [42]. The Kosambi mapping function was used to convert recombination frequency into map distance in centiMorgan (cM). A minimal logarithm-of-the-odds (LOD) score of 4.0, a recombination frequency threshold of 0.325, and a ripple value 1.0 were employed to determine linkage groups (LGs) and marker orders. For each LG, the marker order was determined after three or more runs of the three-round regression algorithm, in which problematic markers, especially those mapped in the third round of the algorithm per run, were removed prior to the next run based on marker quality (exclusively for DArTs) and diagnostic statistics. The LGs were designated according to Brondani et al. [34]. Redundancy of the mapped DArT sequences was estimated with a multiple alignment algorithm implemented in NCBI Blast 2.2.25^+^ (default gapping and an *E*-value of 10^−5^, ftp://ftp.ncbi.nlm.nih.gov/blast/executables/blast+/2.2.25/). Functional annotation for all the mapped markers was performed by BlastX searches against NCBI database of non-redundant protein sequences with a cutoff *E*-value of 10^−5^. Total genome length and map coverage were estimated using the method IV of [43].

For each trait, a value of best linear unbiased prediction (BLUP) was calculated for each full-sib over the six replicates with Proc MIXED in SAS/STAT^®^ 8.0. When necessary, BLUP values of a trait were subjected to Box-Cox transformation for subsequent QTL analysis. The redundant and 3:1 segregating markers were removed from both linkage maps prior to QTL scans. QTLs and their significance were scanned initially using interval mapping (IM) and subsequently by multiple QTL mapping (MQM) under default parameters in software MapQTL 6.0 [44]. Putative QTLs were declared in IM at a specific LOD threshold (between 3.2 and 3.4 for the various traits) calculated from 1,000 permutations at 95% confidence level, and the closest markers were selected as cofactors to carry out MQM analysis. Generally, additional runs of MQM were performed until the QTLs were stably observed.

### Comparative Genomics Analysis

The genetic maps were compared with three earlier maps constructed with SSR and DArT markers in *Eucalyptus*, including the Full map with an *E*. *grandis* × *E*. *urophylla* F_1_ pedigree (GU1) [13], the consensus map with an *E*. *grandis* × *E*. *urophylla* pseudo-backcross F_2_ pedigree (GU2) [12], and the *E*. *globulus* Lighthouse F_2_ map (Glob) [10]. As LGs of all the maps were numbered according to Brondani et al. [34], synteny was defined as common markers that shared the same linkage group. Non-colinearity was determined where order of common markers differed between two maps in an interval more than 1.0 cM [10].

The genetic maps were also compared with the genome sequence of *E*. *grandis* (version 1.1, http://www.phytozome.net/eucalyptus.php). Sequence of each mapped marker was searched with BlastN at an *E*-value of 10^−10^ and the minimum sequence identity of 80.0%. When a marker provided two or more matches, only the highest hit with an *E*-value less than one tenth of the next was assigned. Again, the threshold of 1.0 cM in a genetic map was set for non-colinearity determination.

The QTL regions identified herein were compared with those of the comparable ages mapped on SSR- and DArT-based maps of *E*. *grandis*, *E*. *urophylla* [17], and *E*. *globulus* [18]. For each QTL, the physical distance between flanking markers was deduced by marker sequence alignment with *E*. *grandis* genome, and the QTL approximate physical position (APP) was computed from QTL location relative to the flanking markers.

### Searching for Positional Candidate Genes for QTL Confidence Intervals

Positional candidate genes for QTL confidence intervals were investigated for traits at the latest age, namely, *H*_56_, *D*_56_, and *WD*_56_. Each QTL confidence (95%) interval was aligned to the *E*. *grandis* genome sequence based on conversion of genetic and physical distances between the flanking markers, and a subset of genes were then extracted from the genomic coordinate of the confidence interval (ftp://ftp.jgi-psf.org/pub/compgen/phytozome/v9.0/Egrandis/annotation/Egrandis_201_gene.gff3.gz).

## Results

### Phenotypic Analyses

Mean values, standard deviations, and ranges for growth and wood density traits measured in the mapping population are shown in S1 Table. ANOVA analysis suggested that all traits studied exhibited highly significant variation among the clonal full-sibs (*P* < 0.001; S2 Table), consistent with the highly significant variance component V_G_ (*P* < 0.001; Table 1). Clonal repeatability was high (0.78–0.90; Table 1). Phenotypic correlations between traits were also highly significant (*P* < 0.001; S3 Table). Clonal repeatability in growth traits *H* and *D* (Table 1) and phenotypic correlation between *H* and *D* (S3 Table) appeared to increase with age.

**Table 1:** Genotypic and error variance components (V_G_ and V_E_, respectively) and clonal repeatability (*H* ^2^) for growth and wood density traits of the *E*. *urophylla* × *E*. *tereticornis* mapping population.

Variance component	*H*_10_	*H*_23_	*H*_32_	*H*_44_	*H*_56_	*D*_23_	*D*_32_	*D*_44_	*D*_56_	*WD*_56_
V_G_ ^a^	0.26	1.07	1.52	6.30	9.46	0.87	2.24	5.00	7.64	12.46
V_E_ ^a^	0.43	1.21	1.75	6.01	6.50	1.37	2.38	4.96	6.06	10.41
*H* ^2^ (SE)	0.78 (0.07)	0.84 (0.06)	0.84 (0.06)	0.86 (0.05)	0.90 (0.05)	0.79 (0.07)	0.85(0.05)	0.86 (0.05)	0.88 (0.06)	0.88 (0.06)

^a^ Both V_G_ and V_E_ estimates for each trait were significant at 0.001 level.

### Dense Genetic Maps

A total of 1,693 markers were generated for the mapping population, including 1,187 DArTs, 181 gSSRs, 234 EST-SSRs, and 91 EST-CAPSs (S4 Table). Of these, 626 (37.0%) and 554 (32.7%) markers showed polymorphic alleles unique for maternal *E*. *urophylla* (Ur) and paternal *E*. *tereticornis* (Te) parents, respectively, while 513 (30.3%) markers showed polymorphic alleles for both parents. A relatively high proportion of markers distorted from the expected Mendelian segregation ratios, e.g. 433 markers (25.6%) segregating aberrantly (*P* < 0.05; S5 Table).

The linkage map constructed for *E*. *urophylla* consisted of 700 markers defining 540 independent loci in 11 LGs (Table 2, S1 Fig and S6 Table). The size of the LGs ranged from 69.3 (Ur_LG1) to 167.7 cM (Ur_LG8), averaging at 109.8 cM per LG. The number of loci per LG varied from 26 (Ur_LG4) to 79 (Ur_LG3). The average interval between loci ranged from 1.3 (Ur_LG3) to 3.3 cM (Ur_LG4 and Ur_LG11), with a general mean of 2.3 cM. The total map length reached 1,208.2 cM, covering 95.8% of the estimated genome size (1,261.4 cM). There were eleven gaps of 10.0 cM or higher in length, with the largest being 20.3 cM on Ur_LG11. Of all the mapped markers, 148 (21.1%) were homologous to known or putative genes and 106 (15.1%) were homologous to predicted, hypothetical, or unknown proteins while 388 (55.4%) produced no significant match (*E* ≤ 10^−5^; S6 Table).

**Table 2:** Summary of the markers mapped in each LG of genetic maps of *E*. *urophylla* (Ur) and *E*. *tereticornis* (Te).

LG	No. gSSR	No. EST-SSR	No. EST-CAPS	No. DArT	Subtotal	No. loci	Length (cM)	Mean interval (SD, cM)	No. gSSR	No. EST-SSR	No. EST-CAPS	No. DArT	No. Subtotal	No. loci	Length (cM)	Mean interval (SD, cM)
	Ur								Te							
1	7	6	1	38	52	41	69.3	1.7 (1.8)	6	6	2	24	38	34	104.9	3.2 (3.2)
2	8	8	4	59	79	52	130.8	2.6 (3.1)	6	14	3	31	54	49	156.0	3.3 (2.9)
3	4	13	2	81	100	79	101.8	1.3 (1.2)	3	10	2	67	82	57	106.4	1.9 (1.7)
4	4	4	1	26	35	26	81.9	3.3 (3.6)	1	1	1	38	41	29	75.5	2.7 (2.3)
5	2	5	0	49	56	43	101.4	2.4 (3.2)	5	6	0	51	62	44	105.0	2.4 (2.2)
6	3	16	10	53	82	69	133.6	2.0 (2.0)	4	9	5	16	34	30	110.3	3.8 (3.2)
7	4	4	2	46	56	45	112.1	2.5 (3.8)	1	7	1	48	57	44	102.2	2.4 (2.3)
8	6	12	4	77	99	69	167.7	2.5 (2.1)	5	16	5	52	78	69	168.4	2.5 (2.2)
9	3	7	5	29	44	34	97.1	2.9 (2.6)	3	6	1	19	29	26	113.9	4.6 (3.6)
10	4	8	3	38	53	46	97.2	2.2 (1.9)	3	13	1	29	46	41	98.3	2.5 (2.0)
11	5	8	1	30	44	36	115.3	3.3 (3.7)	4	8	3	49	64	48	100.5	2.1 (2.4)
Subtotal	50	91	33	526					41	96	24	424				
Total					700	540	1,208.2	2.3 (2.6)					585	471	1,241.4	2.7 (2.6)

The linkage map of *E*. *tereticornis* consisted of 585 markers (92 being common and syntenic with Ur map) defining 471 unique loci across 11 LGs, with a total length of 1,241.4 cM and a mean interval of 2.7 cM between loci (Table 2, S1 Fig and S7 Table). The map coverage was estimated to be 95.2%. The LGs ranged from 75.5 (Te_LG4) to 168.4 cM (Te_LG8) in size, averaging at 112.9 cM per LG. The number of loci per LG varied from 26 (Te_LG9) to 69 (Te_LG8), and the average interval between loci per LG ranged from 1.9 (Te_LG3) to 4.6 cM (Te_LG9). Nine gaps were 10.0 cM or higher, with the maximum of 16.9 cM on Te_LG2. Of all the mapped markers, 138 (23.6%) were homologous to known or putative genes and 59 (10.1%) were homologous to predicted, hypothetical, or unknown proteins while 354 (60.5%) produced no significant match (*E* ≤ 10^−5^; S7 Table).

DArT sequence redundancy was relatively high, that is, 29.8% (142/476) and 32.3% (126/390) of the mapped DArTs being redundant in Ur and Te, respectively, at an identity threshold of 80.0% (data available on requrest). Accordingly, clusters of DArT markers were observed in both maps. For instance, there were six and one major DArT clusters in Ur and Te maps, respectively, in which five or more markers were mapped within 0.01 cM and their sequence identity ranged from 31.9% to 99.5% (S8 Table). Those markers with extremely low sequence identities, say, less than 50.0%, may not represent the same locus. Moreover, pronounced clustering of distorted markers was also identified in several LGs of both maps, e.g. Ur_LG5, Te_LG3, and Te_LG8 (S1 Fig).

There were a total of 92 common and syntenic markers between Ur and Te maps (S1 Fig, S6 Table and S7 Table). Each of Ur LGs could be co-aligned with its counterpart in Te or *vice versa*, with two (LG5) to eighteen (LG3) markers shared by each pair of LGs. Of these syntenic markers, 83 (90.2%) showed colinearity in map position, while the rest nine (9.8%) were non-colinear as compared with the alternative LG, including seven and one cases of single- and two-marker rearrangements, respectively.

### QTL Analyses

**Fig 1:**
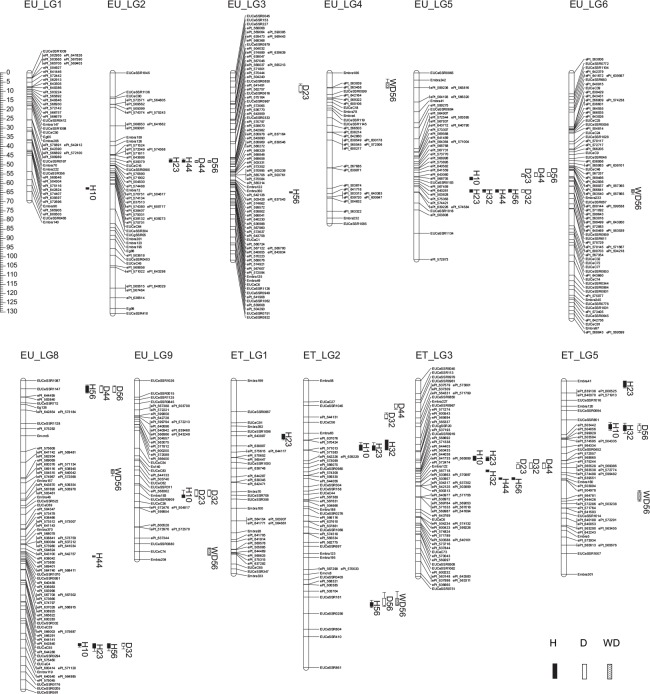
The 31 and 22 QTLs detected in *E*. *urophylla* (Ur) and *E*. *tereticornis* (Te), respectively. Bars and lines represent 1- and 2-logarithm-of-the-odds (LOD) ratio support intervals, respectively. For each QTL, the LOD peak position and 1-LOD (approximately 95%) confidence interval are also indicated in Table 3.

Trait BLUP values for the clonal full-sibs are shown in S9 Table. In total, 31 and 22 QTLs were detected for all the traits across nine LGs in Ur and four LGs in Te, respectively (Fig 1 and Table 3). Twelve regions (six in each of Ur and Te) influenced multiple traits, reducing the total non-overlapping QTL regions to 27 (15 in Ur and 12 in Te). One to four QTLs were identified for each trait on either of the maps, ranging in LOD score from 3.4 (*D*_32_ on Te_LG2) to 7.1 (*H*_10_ on Te_LG3). These QTLs explained between 8.3% (*D*_56_ on Ur_LG5) and 18.9% (*D*_32_ on Ur_LG5) of the total phenotypic variation in the respective traits. For a given trait, QTLs were located on separate LGs of either Ur or Te map, with only one exception being two QTLs for *H*_56_ on Ur_LG8. The QTLs located within homologous LGs between Ur and Te appeared to represent discrete genomic loci regarding their relative positions on genetic maps (Table 3) and physical distance on *E*. *grandis* main scaffolds (data available on request). Wood density QTLs were independent to growth QTLs, except for one coincident QTL region for *WD*_56_, *H*_56_, and *D*_56_ on Te_LG2 (Fig 1, Table 3 and S2 Fig). In most cases, consistent QTLs were identified for height and/or diameter variables at multiple ages, reflecting the relatively strong correlation of growth traits. Also, such consistent QTLs may indicate the stable QTL expression for height or diameter over years, though the magnitude varied with age, e.g. the coincident QTL on Ur_LG5 explaining 10.5–18.3% of the phenotypic variation for height over ages of 23, 32, 44, and 56 months (Table 3). However, QTLs for height or diameter that were expressed at only one age were identified, including those for *H*_10_ on Ur_LGs 1 and 5, *D*_23_ and *H*_56_ on Ur_LG3, and *H*_44_ on Ur_LG8 in Ur as well as *H*_23_ on Te_LGs 1 and 5, *D*_32_ and *D*_44_ on Te_LG2, and *H*_32_ on Te_LG3 in Te (Fig 1 and Table 3).

**Table 3:** QTLs for growth and wood density detected in *E*. *urophylla* (Ur) and *E*. *tereticornis* (Te).

Trait	LG	Position (cM)	LOD	Confidence interval (cM)^a^	Variance explained (%)	LG	Position (cM)	LOD	Confidence interval (cM)^a^	Variance explained (%)
	Ur					Te				
*H*_10_	1	62.8	5.3	62.3–63.3	11.2	2	37.6	4.7	34.0–37.7	11.7
	5	57.7	6.8	56.6–58.9	17.4	3	41.8	7.1	40.7–43.4	18.1
	8	143.4	5.0	142.9–144.8	11.5	5	24.9	4.3	23.3–26.3	9.8
	9	62.9	4.9	59.5–63.6	11.6					
*H*_23_	2	48.0	3.5	47.6–48.4	9.2	1	29.9	4.0	29.2–31.6	9.1
	5	63.9	5.3	63.4–64.8	14.0	2	37.6	3.7	35.9–37.7	8.4
	8	143.6	3.5	143.3–144.6	8.9	3	42.9	6.7	41.1–43.4	16.0
						5	2.0	4.6	0.0–3.6	10.3
*H*_32_	5	63.7	5.8	63.4–64.8	18.3	2	34.7	3.6	32.2–37.2	10.2
						3	49.1	3.6	48.5–49.4	10.5
						5	25.4	3.5	24.6–26.3	9.2
*H*_44_	2	48.2	3.5	47.7–48.5	9.4	3	53.0	4.1	52.8–53.5	13.2
	5	63.9	4.4	63.5–64.8	11.9					
	8	143.8	3.8	143.4–144.4	9.0					
*H*_56_	3	63.4	4.4	63.2–63.5	8.4	2	121.7	3.7	120.9–122.5	10.0
	5	63.9	4.4	63.4–64.8	10.5	3	53.0	3.6	52.8–53.5	11.0
	8	5.4	3.8	3.4–6.0	9.3					
	8	143.6	3.6	143.2–144.5	9.3					
*D*_23_	3	7.7	4.3	6.6–10.8	10.8	3	47.5	3.9	45.8–47.7	11.9
	5	56.2	6.5	54.2–56.6	17.1					
	9	60.3	4.5	59.4–62.8	9.7					
*D*_32_	5	64.1	6.0	61.9–62.4	18.9	2	19.7	3.4	19.0–20.2	9.9
	8	143.4	3.5	143.1–143.9	9.4	3	46.8	4.8	44.6–47.7	14.3
	9	60.6	4.0	59.6–62.7	10.6					
*D*_44_	2	48.5	4.8	47.6–48.6	10.8	2	13.3	3.7	12.8–14.6	11.0
	5	55.7	4.9	54.2–56.6	11.4	3	47.4	4.0	44.9–47.7	11.8
	8	5.5	4.2	3.0–6.3	9.4					
*D*_56_	2	48.5	4.3	47.7–48.6	10.9	2	120.7	4.2	117.8–122.2	12.7
	5	55.6	3.6	54.6–56.3	8.3	5	25.5	4.1	24.2–26.9	13.0
	8	5.4	5.4	2.7–6.4	12.7					
*WD*_56_	4	7.9	4.4	4.5–8.2	10.9	2	120.8	3.9	118.7–121.8	9.6
	6	64.1	5.0	63.4–65.9	10.4	5	62.4	6.4	58.9–65.3	17.2
	8	49.2	5.0	48.2–51.5	11.2					
	9	93.9	4.0	91.3–94.6	9.5					

^a^ 1-logarithm-of-the-odds (LOD) ratio support interval.

### Comparative Genomics Analysis

**Fig 2:**
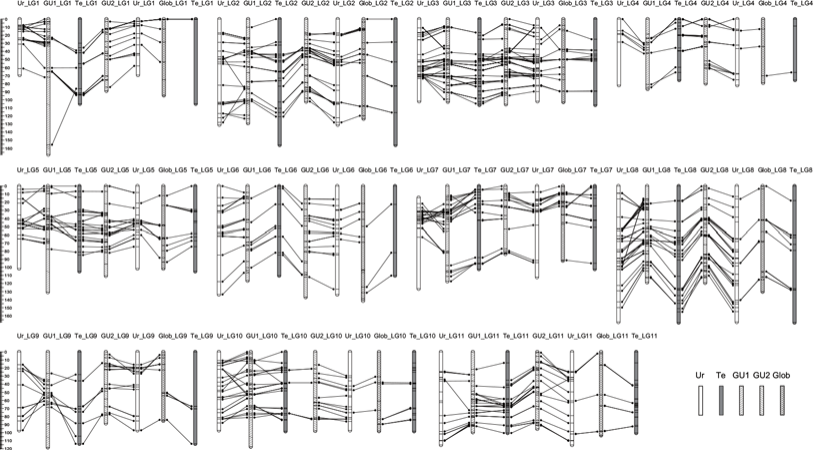
Synteny and colinearity of *E*. *urophylla* (Ur) and *E*. *tereticornis* (Te) maps with prior DArT-based genetic maps of *Eucalyptus*. The prior maps include *E*. *grandis* × *E*. *urophylla* F_1_ Full map (GU1) [13], *E*. *grandis* × *E*. *urophylla* pseudo-backcross F_2_ consensus map (GU2) [12] and *E*. *globulus* Lighthouse F_2_ map (Glob) [10]. Besides top and bottom markers for each linkage group, only positions of common markers between maps are shown.

When compared with the SSR- and DArT-based linkage maps reported earlier for *Eucalyptus*, the maternal Ur map demonstrated a higher level of similarity in intra-section comparison than inter-section comparison (Fig 2 and S10 Table). For instance, 285 and 264 markers were shared in Ur map with GU1 map [13] and GU2 map [12], respectively, within the same section *Latoangulatae*, whereas only 140 markers were in common with Glob map (section *Maidenaria*) [10]. Of the common markers, four (1.4%), eight (3.0%), and eight (5.7%) were non-syntenic, and 76 (27.0%), 37 (14.3%), and 12 (9.1%) were non-colinear to GU1, GU2, and Glob maps, respectively. On the other hand, the map of paternal Te (section *Exsertaria*) had less common markers, namely, 212, 216, and 91 shared with GU1, GU2, and Glob maps, respectively, including 6 (2.8%), 9 (4.2%), and 6 (6.6%) being non-syntenic as well as 45 (21.8%), 22 (10.6%), and 3 (3.5%) non-colinear to the respective maps (S11 Table). For all the comparisons, most non-colinear markers extended across small distance though a few non-colinear occurrences spanned over larger map length (e.g. the top and second markers on Ur_LG3 relative to GU1, GU2, or Glob; Fig 2). Overall, synteny and colinearity were well conserved among the three *Eucalyptus* sections. In addition, both Ur and Te maps had a large number of unique markers as compared to those earlier maps, e.g. a total of 257 and 265 markers being exclusive to GU1, GU2, and Glob maps, respectively (S12 and S13 Tables).

**Fig 3:**
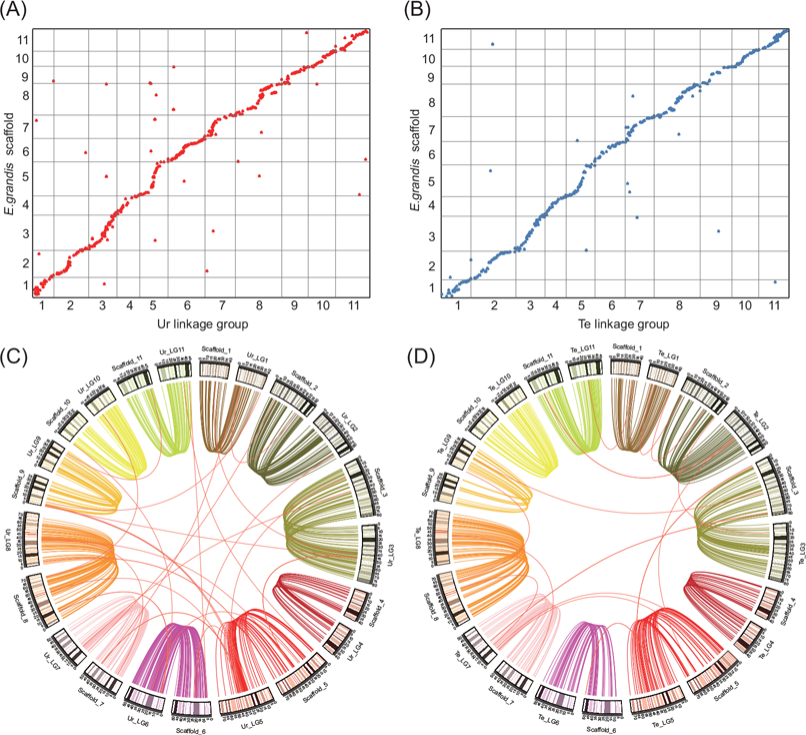
Syntenic relationships of individual linkage groups (LGs) of *E*. *urophylla* (A, C) and *E*. *tereticornis* (B, D) with *E*. *grandis* genome sequence. In (C) and (D), each curve represents a similarity match between a LG and an *E*. *grandis* scaffold, with each thick orange curve showing a case of non-synteny and the different colours reflecting different LGs and the corresponding scaffolds.

A set of 639 and 544 mapped markers from the Ur and Te maps, respectively, could be aligned to *E*. *grandis* genome assembly v1.1 with the exception of those markers either unavailable in nucleotide sequence (55 unique in Ur, 31 unique in Te, and three in common) or with no hit (two unique in Ur, six unique in Te, and one in common; S14 Table). A high level of synteny and colinearity was observed between both of our maps and *E*. *grandis* genome. All the LGs could be aligned to the 11 main scaffolds (Fig 3A, 3B, 3C and 3D). Of those aligned markers, 32 (5.0%) and 173 (27.1%) markers in Ur and 24 (4.4%) and 98 (18.0%) in Te were non-syntenic and non-colinear, respectively. The Ur and Te maps covered 588.5 (97.1%) and 593.7 Mbp (97.7%) of the *E*. *grandis* genome (605.8 Mb), and the mean physical distance per cM was 487.1 and 478.2 kb/cM in Ur and Te, respectively, varying with LG from 337.1 (Te_LG9) to 784.0 kb/cM (Ur_LG3; S14 Table).

QTL comparison was conducted for the closest ages across similar studies using SSR and DArT markers in *Eucalyptus*. None of the QTLs for *D*_56_ or *WD*_56_ was common in APP with those of *E*. *globulus* (age 7) [18], *E*. *grandis* or *E*. *urophylla* (age 4) [17], demonstrating the novelty of these QTLs (S15 Table).

### Positional Candidate Genes for QTL Confidence Intervals

For traits *H*_56_, *D*_56_, and *WD*_56_, the number of positional candidate genes within a QTL confidence interval ranged from 11 (*H*_56_ on Ur_LG3) to 585 (*WD*_56_ on Te_LG5) depending on QTL (S16 and S17 Tables). The total number of positional candidate genes was 255, 577, and 1185, including 83, 253, and 437 in *E*. *urophylla* and 172, 324, and 748 in *E*. *tereticornis*, for *H*_56_, *D*_56_, and *WD*_56_, respectively. Functional annotation showed that 95 (37.3%), 157 (27.2%), and 351 (29.6%) of the positional candidate genes were homologous to known or putative genes, and 136 (53.3%), 371 (64.3%), and 727 (61.4%) corresponded to predicted, hypothetical, unnamed, or uncharacterized proteins while 24 (9.4%), 49 (8.5%), and 107 (9.0%) produced no significant match (*E* ≤ 10^−5^; S16 and S17 Tables) in *H*_56_, *D*_56_, and *WD*_56_, respectively. Moreover, some known genes involved in cell expansion, starch accumulation, leaf resistance, and/or programmed cell death appear to affect growth, and some genes participanting in phenylpropanoid and lignin biosynthesis pathway seem to influence wood density (see Discussion below).

## Discussion

### Dense Genetic Maps for the Important Tree Genus *Eucalyptus*

The *E*. *urophylla* and *E*. *tereticornis* dense maps reported here make a significant contribution to eucalypt genomics resources. The *E*. *tereticornis* map is the first dense map for the important section *Exsertaria*. Both maps contain unique markers that have never been mapped in any prior maps, such as the newly developed EST-SSRs and EST-CAPSs. In total, 1,193 markers were linked in both of our maps (700 in *E*. *urophylla* and 585 in *E*. *tereticornis* including 92 common ones). When compared with prior DArT-based maps in *Eucalyptus*, though the total number of markers mapped here is less than that of the consesus map using two backcrosses between *E*. *grandis* and *E*. *urophylla* (2,290 markers) [12], it is comparable to those of consensus maps based on single inter- (e.g. 1,029 markers for *E*. *grandis* × *E*. *urophylla* F_1_ framework map) [13] or intra-specific crosses (e.g. 1,060 and 569 markers for *E*. *globulus* F_1_ FAM4 and F_2_ LH maps, respectively) [10]. This indicates the relatively high level of polymorphisms in our mapping population. Also, the total map length is similar to other DArT-based eucalypt maps published, e.g. 1,107.6 cM for *E*. *urophylla* BC map [12] and 1,303.9 cM for GU1 map [13], suggesting the similarity in parental recombination rates. On the other hand, considering the markers common with one or more of the prior maps (443 in *E*. *urophylla* and 320 in *E*. *tereticornis*; S10 and S11 Tables), all the dense maps could be merged to form an ultra-dense composite map for *Eucalyptus*. Moreover, our maps were enriched with newly developed EST-SSR and EST-CAPS markers (124 in *E*. *urophylla* and 120 in *E*. *tereticornis* including 46 common ones) that were distributed throughout the 11 LGs. This set of gene-derived markers represents the first step towards development of a sequence-tagged site based transcript map of the *Eucalyptus* genome.

DArT clustering was observed in both of our maps. This may be attributable to DArT sequence redundancy [45], apart from intrinsic recombination reduction [46] and local chromosomal rearrangement [47]. Similar observations have also been reported in other plants including eucalypts [10]. Also, the dominance nature of DArT markers has consequences in their predictive ability for linkage, allele frequency, and marker-trait association [48]. It is thus necessary to intergrate more non-redundant co-dominant markers into the current DArT-dominating maps. Recently, an Infinium^®^ chip with more than 60,000 SNPs has been developed in *Eucalyptus* [49]. In addition, advances in next generation sequencing have offered the feasibility of genotyping by sequencing for genetic mapping of heterozygous genomes, e.g. maize [50]. All these advances could be highly conducive for further improvements in genetic mapping in *Eucalyptus*.

### Comparative Genome Mapping in *Eucalyptus*

The high levels of genome synteny and colinearity presented here corroborate the findings of Hudson et al. [10] who made intra- and inter-sectional comparisons between DArT-based maps of *E*. *globulus*, *E*. *grandis*, and *E*. *urophylla*. In comparisons within section *Latoangulatae*, 98.6% and 97.0% of common markers were syntenic in our *E*. *urophylla* map to the GU1 [13] and GU2 [12] maps, with 27.0% and 14.3% being no-colinear, respectively (S10 Table). In inter-sectional comparison, slightly higher degrees of non-synteny (2.8–6.6%; S10 and S11 Tables) were observed in our *E*. *urophylla* and *E*. *tereticornis* maps, with 3.5–21.8% of syntenic markers being non-colinear. Similarly, Hudson et al. [10] found map non-synteny percentages of 0.8%, 1.3%, and 6.6% and non-colinearity of 4.7%, 6.8%, and 6.3% within section *Latoangulatae*, within *Maidenaria*, and between the two sections, respectively. Such non-synteny and non-colinearity could result from multiple factors, such as intra- and inter-chromosomal locus duplication, genome rearrangement, transposon-mediated marker transposition, discrepancy in recombination rate among different genomic regions, small mapping population size, compromised marker orders in a consensus map, missing data, and genotyping errors [10,42,46]. Nevertheless, the non-synteny level agrees well with taxonomic relationships. As sections *Exsertaria* and *Latoangulatae* are very close and should probably be combined into a single section [51], it is not unexpected that their synteny level (*E*. *tereticornis* vs GU1 or GU2) is comparable to that within *Latoangulatae* (*E*. *urophylla* vs GU1 or GU2) and both sections have a similarly less synteny with *Maidenaria* (*E*. *globulus*). However, the proportion of non-colinear markers appears to decrease with taxonomic distance, e.g. 27.0% for *E*. *urophylla* vs GU1 but 9.1% for *E*. *urophylla* vs Glob (S10 Table). In addition to the potential factors described above, this may be attributed to the markedly smaller number of syntenic markers in Glob relative to GU1 and GU2, resulting in larger map intervals for co-linearity analysis where occurrence of marker rearrangements could be less possible.

High levels of synteny and colinearity were also revealed when compared to *E*. *grandis* genome assembly. Of all the mapped markers, only three (0.5%) in *E*. *urophylla* and seven (1.3%) in *E*. *tereticornis* did not have a significant match to *E*. *grandis* genome (S14 Table). Such missing matches could imply the presence of specific sequences that may not have been assembled in *E*. *grandis* genome or represent novel loci evolving in the specific species. Thus, the maps presented here would add independent information to the current *E*. *grandis* genome. Also, 32 (5.0%) and 173 (27.1%) markers in the Ur map and 24 (4.4%) and 98 (18.0%) in the Te map were non-syntenic and non-colinear, respectively, to the *E*. *grandis* genome (S14 Table). Considering that *E*. *grandis* genome has more tandem duplicates than other plants [28], a marker may be aligned to an alternative physical region of a duplicate and thus can give rise to non-synteny and non-colinearity. In addition, the mean physical distance per cM was less than the estimate of 513 kb/cM reported by Petroli et al. [13], but substantially higher than some selfing plants like rice (*c*. 244 kb/cM) [52]. This may pose a challenge for map-based cloning, though recombination frequency can vary largely with position along the chromosome [52].

### QTLs and Positional Candidate Genes for Growth and Wood Density

Taking both parents together, the number of QTLs detected for wood density is the same as or very close to that of growth (*D* or *H*) at the same age, as is expected from the similar magnitude of *H* ^2^ estimates (Table 1). The moderate proportion of the phenotypic variance (8.3–18.9%) explained by individual QTLs is generally parallel with those reported previously in trees, e.g. *Eucalyptus* [17,18], *Populus* [15], and *Pinus* [53]. This indicates the polygenic nature of the traits analyzed. Moreover, growth (*H* or *D*) and wood density QTLs were located in different regions in both parental genomes, except for *D*_56_ and *WD*_56_ on Te_LG2 (Fig 1 and Table 3). Also, in addition to co-locating QTL(s) for growth and wood density, separate QTLs were identified for both trait types in other tree species, such as *E*. *grandis* [54,55] and *Pinus radiata* [53], reflecting the independence of major QTLs affecting the two types of traits despite occasional co-locating loci due to pleiotropic effects and/or tight linkage between QTLs. Furthermore, for highly correlated growth traits, like *H* and *D* at the same age or either variable over two or more ages, consistent QTLs identified may indicate the existence of pleiotropic genes or the stable expression of genes over time. Though stable QTLs between traits or over time are not frequent in forest tree literature, several studies have reported QTL stability, e.g. a QTL in *E*. *globulus* for one-year-old height as well as two- and six-year-old diameters [56], and a number of QTLs overlapping among bud flush, bud set, and height traits and/or among years in *Picea glauca* [16]. Even so, no QTL was significant in our study over all ages of growth and the observed number of co-locating loci was lower than expected. On the other hand, given the relatively small population size used here, the number of QTLs for a specific trait may have been underestimated and their phenotypic effects overestimated [56–58]. In forest trees, due to mostly the limitations in manageable population size, QTL mapping has generally been carried out in small populations and much has been learned about complex trait architecture such as the number, position, and size of effects of QTLs [21]. For instance, families each with around 100 genotypes have been recently used for QTL detection in *Populus* [59] and *Eucalyptus* [18]. Nonetheless, larger populations of the same cross or different pedigrees will be useful to validate the effects of QTLs identified in the present study and detect additional QTLs.

We failed to detect QTLs common with those mapped on DArT-based maps of *E*. *grandis*, *E*. *urophylla* [17] and *E*. *globulus* [18]. This may be due to the lack of segregation at QTLs in alternative populations and/or due to differences in environment and developmental stage [18,21]. Meanwhile, those novel QTLs detected here would add significantly to the list of genomic regions affecting growth and wood density in *Eucalyptus* trees. Moreover, the relatively high *H* ^2^ values (Table 1) suggest that genetic gain could be obtained through selection in growth and wood property traits. As traditional phenotypic selection is laborious and time-consuming, marker assisted selection (MAS) provides an efficient method for selecting genotypes of interest. In this respect, the identification of novel QTLs would be valuable for future MAS programs.

The released *E*. *grandis* genome sequence allows for investigating positional candidate genes within each QTL confidence interval *in silico*. A number of positional candidate genes identified herein had known functions. For *H*_56_ and *D*_56_, some candidate genes involved in cell expansion, starch accumulation, leaf resistance, and/or programmed cell death appear to affect growth. For example, wall associated kinase (Eucgr.E03106.1; S16 and S17 Tables) and xyloglucan endotransglycosylase (Eucgr.C02203.1; S17 Table) are required for cell expansion [60,61], glucose-1-phosphate adenylyltransferase (Eucgr.B01108.1; S16 Table) functions during starch accumulation and carbon allocation [62], stem-specific protein TSJT1 (Eucgr.B01123.1; S16 Table) expresses differently during H_2_O_2_-induced programmed cell death [63], and stearoyl-ACP desaturase (Eucgr.H00294.1−00307.1 except 00302.1−00304.1; S16 Table) modulates the activation of plant defense signaling pathways such that the mutant plants of a stearoyl-ACP desaturase gene *SSI2* are dwarfed [64]. For *WD*_56_, lignin seems to play an important role in trait formation as a range of candidate genes are participants in phenylpropanoid and lignin biosynthesis pathway, such as lignin-forming anionic peroxidase precursor (Eucgr.I02714.1; S16 Table), cinnamyl alcohol dehydrogenase (Eucgr.E02559.1; S17 Table), hydroxycinnamoyl-CoA shikimate/quinate hydroxycinnamoyltransferase (Eucgr.E02701.1; S17 Table), and caffeic acid 3-O-methyltransferase (Eucgr.E03146.1 and 03148.1; S17 Table). Also, several genes were identified earlier to link lignin traits in *Populus* [24], e.g. NAC domain-containing protein (Eucgr.E03226.1; S17 Table), glycosyltransferase (Eucgr.H00262.1 and E02950.1; S16 and S17 Tables, respectively), and cytochrome P450 (Eucgr.E02618.1 and 02619.1; S17 Table). In addition, F-box family protein may affect wood density in *Eucalyptus* as shown in Kullan et al. [17] and this study (Eucgr.E02620.1; S17 Table).

## Conclusions

Dense genetic maps of *E*. *urophylla* and *E*. *tereticornis* were constructed using genomic SSR, EST-SSR, EST-CAPS, and DArT markers. As compared with the dense maps of *Eucalyptus* reported earlier and the *E*. *grandis* genome sequence released recently, high levels of genomic synteny and colinearity were revealed for both of our maps, which would assist in composite map construction and cross-species QTL mapping in the important hardwood genus *Eucalyptus*. Novel QTLs associated with growth and wood density were detected on the maps, adding promising targets for future MAS programs. Positional candidate genes for each 56-month-old QTL confidence interval were identified, in which relatively low numbers of positional candidate genes were found for certain QTLs. This will facilitate functional characterization of specific causative genes for tree growth and wood density. Larger populations are under development to increase the statistic power of QTL mapping and target more economic traits. As *Eucalyptus* genome serves as the pivotal representative of the large dicotyledonous woody family Myrtaceae [23], results from this study would provide a valuable resource for genomics studies and directed breeding of other closely related genera in the family.

## Supporting Information

S1 Fig Genetic maps of *Eucalyptus urophylla* (Ur) and *E*. *tereticornis* (Te). Linkage groups (LGs) are designated according to Brondani et al. [34]. Accumulated map distances (cM) and locus names are presented to the left and right of each LG, respectively. Common markers in both maps are connected by a line. Marker segregation distortions are marked with * (*P* ≤ 0.05), ** (*P* ≤ 0.01), and *** (*P* ≤ 0.001).
(PDF)

S2 Fig QTL analysis for 56-month-old height (*H*_56_), diameter at breast height (*D*_56_), and wood density (*WD*_56_) in *Eucalyptus urophylla* (Ur) and *E*. *tereticornis* (Te). Each linkage group is the same as shown in S1 Fig A black dashed line indicates the LOD threshold (usually being 3.4 and 3.3 or 3.2 in a few cases) used for declaring a QTL in software MapQTL 6.0 [44].
(PDF)

S1 Table Means (standard deviations, SD) and ranges for height (*H*) at 10, 23, 32, 44, and 56 months of age (*H*_10_, *H*_23_, *H*_32_, *H*_44_, and *H*_56_, respectively), diameter at breast height (*D*) at 23, 32, 44, and 56 months (*D*_23_, *D*_32_, *D*_44_, and *D*_56_, respectively), and 56-month-old wood density (*WD*_56_) measured in the *E*. *urophylla* × *E*. *tereticornis* mapping population. (DOC)

S2 Table Mean squares from ANOVA for growth and wood density of the *E*. *urophylla* × *E*. *tereticornis* mapping population. (DOC)

S3 Table Pearson correlation coefficients between traits of the mapping population. (DOC)

S4 Table Number of diversity arrays technology (DArT), genomic simple sequence repeats (gSSR), expressed sequence tag-derived SSR (EST-SSR), and EST-derived cleaved amplified polymorphic sequence (CAPS) markers segregating in the *E*. *urophylla* × *E*. *tereticornis* mapping population. (DOC)

S5 Table Number of markers showing segregation distortion at various significant levels in the *E*. *urophylla* × *E*. *tereticornis* mapping population. (DOC)

S6 Table Markers mapped on the genetic map of maternal *E*. *urophylla* (Ur) as well as their segregation, functional annotation, and alignment with *E*. *grandis* genome sequence. (XLS)

S7 Table Markers mapped on the genetic map of paternal *E*. *tereticornis* (Te) as well as their segregation, functional annotation, and alignment with *E*. *grandis* genome sequence. (XLS)

S8 Table Sequence identity (%) between DArT markers clustered (with five or more markers within 0.01 cM) on the genetic maps of *E*. *urophylla* (Ur) and *E*. *tereticornis* (Te). (DOC)

S9 Table The best linear unbiased prediction (BLUP) trait values for the clonal full-sibs of the mapping population. (DOC)

S10 Table Numbers of common, non-syntenic, and non-colinear markers in *E*. *urophylla* as compared with prior DArT-based genetic maps of *Eucalyptus*, including *E*. *grandis* × *E*. *urophylla* F_1_ Full map (GU1) [13], *E*. *grandis* × *E*. *urophylla* pseudo-backcross F_2_ consensus map (GU2) [12], and *E*. *globulus* Lighthouse F_2_ map (Glob) [10]. (DOC)

S11 Table Numbers of common, non-syntenic, and non-colinear markers in *E*. *tereticornis* as compared with prior DArT-based genetic maps of *Eucalyptus*, including *E*. *grandis* × *E*. *urophylla* F_1_ Full map (GU1) [13], *E*. *grandis* × *E*. *urophylla* pseudo-backcross F_2_ consensus map (GU2) [12], and *E*. *globulus* Lighthouse F_2_ map (Glob) [10]. (DOC)

S12 Table Unique markers in *E*. *urophylla* (Ur) genetic map as compared with prior SSR- and DArT-based genetic maps of *Eucalyptus*, including *E*. *grandis* × *E*. *urophylla* F_1_ Full map (GU1) [13], *E*. *grandis* × *E*. *urophylla* pseudo-backcross F_2_ consensus map (GU2) [12], and *E*. *globulus* Lighthouse F_2_ map (Glob) [10]. (DOC)

S13 Table Unique markers in *E*. *tereticornis* (Te) genetic map as compared with prior SSR- and DArT-based genetic maps of *Eucalyptus*, including *E*. *grandis* × *E*. *urophylla* F_1_ Full map (GU1) [13], *E*. *grandis* × *E*. *urophylla* pseudo-backcross F_2_ consensus map (GU2) [12], and *E*. *globulus* Lighthouse F_2_ map (Glob) [10]. (DOC)

S14 Table Statistics for alignment of *E*. *urophylla* and *E*. *tereticornis* genetic maps with *E*. *grandis* genome sequence V1.1. (DOC)

S15 Table Comparison of QTLs detected on the homologous linkage groups across SSR- and DArT-based studies in *Eucalyptus*. (DOC)

S16 Table Positional candidate genes (PCGs) within each QTL confidence interval for 56-month-old height (*H*_56_), diameter at breast height (*D*_56_), and wood density (*WD*_56_) in *E*. *urophylla* (Ur) as aligned with *E*. *grandis* genome sequence. (XLS)

S17 Table Positional candidate genes (PCGs) within each QTL confidence interval for 56-month-old height (*H*_56_), diameter at breast height (*D*_56_), and wood density (*WD*_56_) in *E*. *tereticornis* (Te) as aligned with *E*. *grandis* genome sequence. (XLS)

## References

[1] Wu J, Maehara T, Shimokawa T, Yamamoto S, Harada C, Takazaki Y, et al A comprehensive rice transcript map containing 6591 expressed sequence tag sites. Plant Cell. 2002; 14: 525–535. 11910001 10.1105/tpc.010274 PMC150576

[2] Ganal MW, Durstewitz G, Polley A, Bérard A, Buckler ES, Charcosset A, et al A large maize (*Zea mays* L.) SNP genotyping array: development and germplasm genotyping, and genetic mapping to compare with the B73 reference genome. PLoS One. 2011; 6: e28334 10.1371/journal.pone.0028334 22174790 PMC3234264

[3] Ashikari M, Matsuoka M. Identification, isolation and pyramiding of quantitative trait loci for rice breeding. Trends Plant Sci. 2006; 11: 344–360. 16769240 10.1016/j.tplants.2006.05.008

[4] Miura K, Ashikari M, Matsuoka M. The role of QTLs in the breeding of high-yielding rice. Trends Plant Sci. 2011; 16: 319–326. 10.1016/j.tplants.2011.02.009 21429786

[5] Gao Z-Y, Zhao S-C, He W-M, Guo L-B, Peng Y-L, Wang J-J, et al Dissecting yield-associated loci in super hybrid rice by resequencing recombinant inbred lines and improving parental genome sequences. Proc Natl Acad Sci USA. 2013; 107: 14492–14497. 10.1073/pnas.1306579110 PMC3761582 23940322

[6] Drost DR, Novaes E, Boaventura-Novaes C, Benedict CI, Brown RS, Yin T, et al A microarray-based genotyping and genetic mapping approach for highly heterozygous outcrossing species enables localization of a large fraction of the unassembled *Populus trichocarpa* genome sequence. Plant J. 2009; 58: 1054–1067. 10.1111/j.1365-313X.2009.03828.x 19220791

[7] Kang B-Y, Mann IK, Major JE, Rajora OP. Near-saturated and complete genetic linkage map of black spruce (*Picea mariana*). BMC Genomics. 2010; 11: 515 10.1186/1471-2164-11-515 20868486 PMC2997009

[8] Moriguchi Y, Ujino-Ihara T, Uchiyama K, Futamura N, Saito M, Ueno S, et al The construction of a high-density linkage map for identifying SNP markers that are tightly linked to a nuclear-recessive major gene for male sterility in *Cryptomeria japonica* D. Don. BMC Genomics. 2012; 13: 95 10.1186/1471-2164-13-95 22424262 PMC3386010

[9] Martínez-García PJ, Stevens KA, Wegrzyn JL, Liechty J, Crepeau M, Langley CH, et al Combination of multipoint maximum likelihood (MML) and regression mapping algorithms to construct a high-density genetic linkage map for loblolly pine (*Pinus taeda* L.). Tree Genet Genomes. 2013; 9: 1529–1535.

[10] Hudson CJ, Kullan ARK, Freeman JS, Faria DA, Grattapaglia D, Kilian A, et al High synteny and colinearity among *Eucalyptus* genomes revealed by high-density comparative genetic mapping. Tree Genet Genomes. 2012; 8: 339–352.

[11] Neves LG, Mamani EMC, Alfenas AC, Kirst M, Grattapaglia D. A high-density transcript linkage map with 1,845 expressed genes positioned by microarray-based Single Feature Polymorphisms (SFP) in *Eucalyptus* . BMC Genomics. 2011; 12: 189 10.1186/1471-2164-12-189 21492453 PMC3090358

[12] Kullan ARK, van Dyk MM, Jones N, Kanzler A, Bayley A, Myburg AA. 2012. High-density genetic linkage maps with over 2,400 sequence-anchored DArT markers for genetic dissection in an F_2_ pseudo-backcross of *Eucalyptus grandis* × *E*. *urophylla* . Tree Genet Genomes. 2012; 8: 163–175.

[13] Petroli CD, Sansaloni CP, Carling J, Stean DA, Vaillancourt RE, Myburg AA, et al Genomic characterization of DArT markers based on high-density linkage analysis and physical mapping to the *Eucalyptus* genome. PLoS One. 2012; 7: e44684 10.1371/journal.pone.0044684 22984541 PMC3439404

[14] Bartholomé J, Mandrou E, Mabiala A, Jenkins J, Nabihoudine I, Klopp C, et al High-resolution genetic maps of *Eucalyptus* improve *Eucalyptus grandis* genome assembly. New Phytol. 2015; 206: 1283–1296. 10.1111/nph.13150 25385325

[15] Novaes E, Osorio L, Drost DR, Miles BL, Boaventura-Novaes CRD, Benedict C, et al Quantitative genetic analysis of biomass and wood chemistry of *Populus* under different nitrogen levels. New Phytol. 2009; 182: 878–890. 10.1111/j.1469-8137.2009.02785.x 19291008

[16] Pelgas B, Bousquet J, Meirmans PM, Ritland K. QTL mapping in white spruce: gene maps and genomic regions underlying adaptive traits across pedigrees, years and environments. BMC Genomics. 2011; 12: 145 10.1186/1471-2164-12-145 21392393 PMC3068112

[17] Kullan ARK, van Dyk MM, Hefer CA, Jones N, Kanzler A, Myburg AA. Genetic dissection of growth, wood basic density and gene expression in interspecific backcrosses of *Eucalyptus grandis* and *E*. *urophylla* . BMC Genetics. 2012; 13: 60 10.1186/1471-2156-13-60 22817272 PMC3416674

[18] Freeman JS, Potts BM, Downes GM, Pilbeam D, Thavamanikumar S, Vaillancourt RE. 2013. Stability of quantitative trait loci for growth and wood properties across multiple pedigrees and environments in *Eucalyptus globulus* . New Phytol. 2013; 198: 1121–1134. 10.1111/nph.12237 23517065

[19] Bartholomé J, Mabiala A, Savelli B, Bert D, Brendel O, Plomion C, et al Genetic architecture of carbon isotope composition and growth in *Eucalyptus* across multiple environments. New Phytol. 2015; 206: 1437–1449. 10.1111/nph.13301 25643911

[20] Grattapaglia D, Plomion C, Kirst M, Sederoff RR. Genomics of growth traits in forest trees. Curr Opin Plant Biol. 2009; 12: 148–156. 10.1016/j.pbi.2008.12.008 19186096

[21] Neale DB, Kremer A. Forest tree genomics: growing resources and applications. Nat Rev Genet. 2011; 12: 111–122. 10.1038/nrg2931 21245829

[22] Grattapaglia D, Kirst M. *Eucalyptus* applied genomics: from gene sequences to breeding tools. New Phytol. 2008; 179: 911–929. 10.1111/j.1469-8137.2008.02503.x 18537893

[23] Grattapaglia D, Vaillancourt RE, Shepherd M, Thumma BR, Foley W, Külheim C, et al Progress in Myrtaceae genetics and genomics: *Eucalyptus* as the pivotal genus. Tree Genet Genomes. 2012; 8: 463–508.

[24] Ranjan P, Yin T, Zhang X, Kalluri UC, Yang X, Jawdy S, et al Bioinformatics-based identification of candidate genes from QTLs associated with cell wall traits in *Populus* . Bioenerg Res. 2010; 3: 172–182.

[25] Monclus R, Leplé J-C, Bastien C, Bert P-F, Villar M, Marron N, et al Integrating genome annotation and QTL position to identify candidate genes for productivity, architecture and water-use efficiency in *Populus* spp. BMC Plant Biol. 2012; 12: 173 10.1186/1471-2229-12-173 23013168 PMC3520807

[26] GIT. Global eucalypt forests map 2009 Lugo: GIT Forestry Consulting SL; 2009.

[27] Grattapaglia D, Bradshaw HD Jr. Nuclear DNA content of commercially important *Eucalyptus* species and hybrids. Can J For Res. 1994; 24: 1074–1078.

[28] Myburg AA, Grattapaglia D, Tuskan GA, Hellsten U, Hayes RD, Grimwood J, et al The genome of *Eucalyptus grandis* . Nature. 2014; 510: 356–362. 10.1038/nature13308 24919147

[29] Yu X, Guo Y, Zhang X, Li F, Weng Q, Li M, et al Integration of EST-CAPS markers into genetic maps of *Eucalyptus urophylla* and *E*. *tereticornis* and their alignment with *E*. *grandis* genome sequence. Silvae Genet. 2012; 61: 247–255.

[30] Weng Q, He X, Li F, Li M, Yu X, Shi J, et al Hybridizing ability and heterosis between *Eucalyptus urophylla* and *E*. *tereticornis* for growth and wood density over two environments. Silvae Genet. 2014; 63: 15–24.

[31] Zhou C, Li F, Weng Q, Yu X, Li M, Gan S. Comparison between direct sequencing and pool-cloning-based sequencing of PCR products in EST-SSR marker development in *Eucalyptus* . Mol Plant Breed. 2010; 8: e1 10.5376/mpb.cn.2010.08.0001

[32] He X, Wang Y, Li F, Weng Q, Li M, Xu L, et al Development of 198 novel EST-derived microsatellites in *Eucalyptus* (Myrtaceae). Am J Bot. 2012; 99: e134–e148. 10.3732/ajb.1100442 22473983

[33] Zhou C, He X, Li F, Weng Q, Yu X, Wang Y, et al Development of 240 novel EST-SSRs in *Eucalyptus* L'Hérit. Mol Breed. 2014; 33: 221–225.

[34] Brondani RPV, Williams ER, Brondani C, Grattapaglia D. A microsatellite-based consensus linkage map for species of *Eucalyptus* and a novel set of 230 microsatellite markers for the genus. BMC Plant Biol. 2006; 6: 20 10.1186/1471-2229-6-20 16995939 PMC1599733

[35] Steane DA, Vaillancourt RE, Russell J, Powell W, Marshall D, Potts BM. Development and characterisation of microsatellite loci in *Eucalyptus globulus* (Myrtaceae). Silvae Genet. 2001; 50: 89–91.

[36] He X, Li F, Shi J, Gan S. Seven genomic SSR markers revealed in *Eucalyptus* by re-sequencing of DNA sequences from GenBank. Silvae Genet. 2011; 60: 92–94.

[37] Byrne M, Marquez-Garcia MI, Uren T, Smith DS, Moran GF. Conservation and genetic diversity of microsatellite loci in the genus *Eucalyptus* . Aust J Bot. 1996; 44: 331–341.

[38] Glaubitz JC, Emebiri LC, Moran GF. Dinucleotide microsatellites from *Eucalyptus sieberi*: inheritance, diversity, and improved scoring of single-base differences. Genome. 2001; 44: 1041–1045. 11768207

[39] Ottewell KM, Donnellan SC, Moran GF, Paton DC. Multiplexed microsatellite markers for the genetic analysis of *Eucalyptus leucoxylon* (Myrtaceae) and their utility for ecological and breeding studies in other *Eucalyptus* species. J Hered. 2005; 96: 445–451. 15843635 10.1093/jhered/esi057

[40] Li F, Gan S. An optimised protocol for fluorescent-dUTP based SSR genotyping and its application to genetic mapping in *Eucalyptus* . Silvae Genet. 2011; 60: 18–25.

[41] van Ooijen JW. JoinMap® 4: Software for the calculation of genetic linkage maps in experimental populations Wageningen: Kyazma BV; 2006.

[42] Hackett CA, Broadfoot LB. Effects of genotyping errors, missing values and segregation distortion in molecular marker data on the construction of linkage maps. Heredity. 2003; 90: 33–38. 12522423 10.1038/sj.hdy.6800173

[43] Chakravarti A, Lasher LA, Reefer JE. A maximum likelihood method for estimating genome length using genetic linkage data. Genetics. 1991; 128: 175–182. 2060775 10.1093/genetics/128.1.175 PMC1204446

[44] van Ooijen JW. MapQTL® 6: Software for the mapping of quantitative trait loci in experimental populations of diploid species Wageningen: Kyazma BV; 2009.

[45] Wenzl P, Li H, Carling J, Zhou M, Raman H, Paul E, et al A high-density consensus map of barley linking DArT markers to SSR, RFLP and STS loci and agricultural traits. BMC Genomics. 2006; 7: 206 10.1186/1471-2164-7-206 16904008 PMC1564146

[46] Raman H, Raman R, Kilian A, Detering F, Long Y, Edwads D, et al A consensus map of rapeseed (*Brassica napus* L.) based on diversity array technology markers: applications in genetic dissection of qualitative and quantitative traits. BMC Genomics. 2013; 14: 277 10.1186/1471-2164-14-277 23617817 PMC3641989

[47] Wight CP, Tinker NA, Kianian SF, Sorrells ME, O'Donoughue LS, Hoffman DL, et al A molecular marker map in 'Kanota' × 'Ogle' hexaploid oat (*Avena* spp.) enhanced by additional markers and a robust framework. Genome. 2003; 46: 28–47. 12669794 10.1139/g02-099

[48] King J, Thomas A, James C, King I, Armstead I. A DArT marker genetic map of perennial ryegrass (*Lolium perenne* L.) integrated with detailed comparative mapping information; comparison with existing DArT marker genetic maps of *Lolium perenne*, *L*. *multiflorum* and *Festuca pratensis* . BMC Genomics. 2013; 14: 437 10.1186/1471-2164-14-437 23819624 PMC3704433

[49] Silva-Junior OB, Faria DA, Grattapaglia D. A flexible multi-species genome-wide 60K SNP chip developed from pooled resequencing of 240 *Eucalyptus* tree genomes across 12 species. New Phytol. 2015; 206: 1527–1540. 10.1111/nph.13322 25684350

[50] Elshire RJ, Glaubitz JC, Sun Q, Poland JA, Kawamoto K, Buckler ES, et al A robust, simple genotyping-by-sequencing (GBS) approach for high diversity species. PLoS One. 2011; 6: e19379 10.1371/journal.pone.0019379 21573248 PMC3087801

[51] Steane DA, Nicolle D, McKinnon GE, Vaillancourt RE, Potts BM. Higher-level relationships among the eucalypts are resolved by ITS-sequence data. Aust Sys Bot. 2002; 15: 49–62.

[52] Chen M, Presting G, Barbazuk WB, Goicoechea JL, Blackmon B, Fang G, et al An integrated physical and genetic map of the rice genome. Plant Cell. 2002; 14: 537–545. 11910002 10.1105/tpc.010485 PMC150577

[53] Devey ME, Carson SD, Nolan MF, Matheson AC, Te Riini C, Hohepa J. QTL associations for density and diameter in *Pinus radiata* and the potential for marker-aided selection. Theor Appl Genet. 2004; 108: 516–524. 14657985 10.1007/s00122-003-1446-2

[54] Grattapaglia D, Bertolucci FLG, Penchel R, Sederoff RR. Genetic mapping of quantitative trait loci controlling growth and wood quality traits in *Eucalyptus grandis* using a maternal half-sib family and RAPD markers. Genetics. 1996; 144: 1205–1214. 8913761 10.1093/genetics/144.3.1205 PMC1207612

[55] Gion J-M, Carouché A, Deweer S, Bedon F, Pichavant F, Charpentier J-P, et al Comprehensive genetic dissection of wood properties in a widely-grown tropical tree: *Eucalyptus* . BMC Genomics. 2011; 12: 301 10.1186/1471-2164-12-301 21651758 PMC3130712

[56] Bundock PC, Potts BM, Vaillancourt RE. Detection and stability of quantitative trait loci (QTL) in *Eucalyptus globulus* . Tree Genet Genomes. 2008; 4: 85–95.

[57] Beavis WD. QTL analysis: power, precision, and accuracy In: Paterson AH, editor. Molecular dissection of complex traits. Boca Raton: CRC Press; 1998 pp. 145–162.

[58] Bartholomé J, Salmon F, Vigneron P, Bouvet J-M, Plomion C, Gion J-M. Plasticity of primary and secondary growth dynamics in *Eucalyptus* hybrids: a quantitative genetics and QTL mapping perspective. BMC Plant Biol. 2013; 13: 120 10.1186/1471-2229-13-120 23978279 PMC3870978

[59] Fabbrini F, Gaudet M, Bastien C, Zaina G, Harfouche A, Beritognolo I, et al Phenotypic plasticity, QTL mapping and genomic characterization of bud set in black poplar. BMC Plant Biol. 2012; 12: 47 10.1186/1471-2229-12-47 22471289 PMC3378457

[60] Wagner TA, Kohorn BD. Wall-associated kinases are expressed throughout plant development and are required for cell expansion. Plant Cell. 2001; 13: 303–318. 11226187 10.1105/tpc.13.2.303 PMC102244

[61] Bourquin V, Nishikubo N, Abe H, Brumer H, Denman S, Eklund M, et al Xyloglucan endotransglycosylases have a function during the formation of secondary cell walls of vascular tissues. Plant Cell. 2002; 14: 3073–3088. 12468728 10.1105/tpc.007773 PMC151203

[62] Wingler A, Fritzius T, Wiemken A, Boller T, Aeschbacher RA. Trehalose induces the ADP-glucose pyrophosphorylase gene, *ApL3*, and starch synthesis in *Arabidopsis* . Plant Physiol. 2000; 124: 105–114. 10982426 10.1104/pp.124.1.105 PMC59126

[63] Vannini C, Marsoni M, Cantara C, de Pinto MC, Locato V, de Gara L, et al The soluble proteome of tobacco Bright Yellow-2 cells undergoing H_2_O_2_-induced programmed cell death. J Exp Bot. 2012; 63: 3137–3155. 10.1093/jxb/ers031 22355080 PMC3350924

[64] Kachroo P, Shanklin J, Shah J, Whittle EJ, Klessig DF. A fatty acid desaturase modulates the activation of defense signaling pathways in plants. Proc Natl Acad Sci USA. 2001; 98: 9448–9453. 11481500 10.1073/pnas.151258398 PMC55441

