# Supplementary material for: Comparative Genomics Analyses Reveal Extensive Chromosome Colinearity and Novel Quantitative Trait Loci in Eucalyptus
Source: PLoS One. 2015 Dec 22;10(12):e0145144. doi: 10.1371/journal.pone.0145144 (PMC4687840; doi:10.1371/journal.pone.0145144)
Supplement: S1 Fig — Linkage groups (LGs) are designated according to Brondani et al. [34]. Accumulated map distances (cM) and locus names are presented to the left and right of each LG, respectively. Common markers in both maps are connected by a line. Marker segregation distortions are marked with * (P ≤ 0.05), ** (P ≤ 0.01), and *** (P ≤ 0.001). (PDF) [file pone.0145144.s001.pdf]

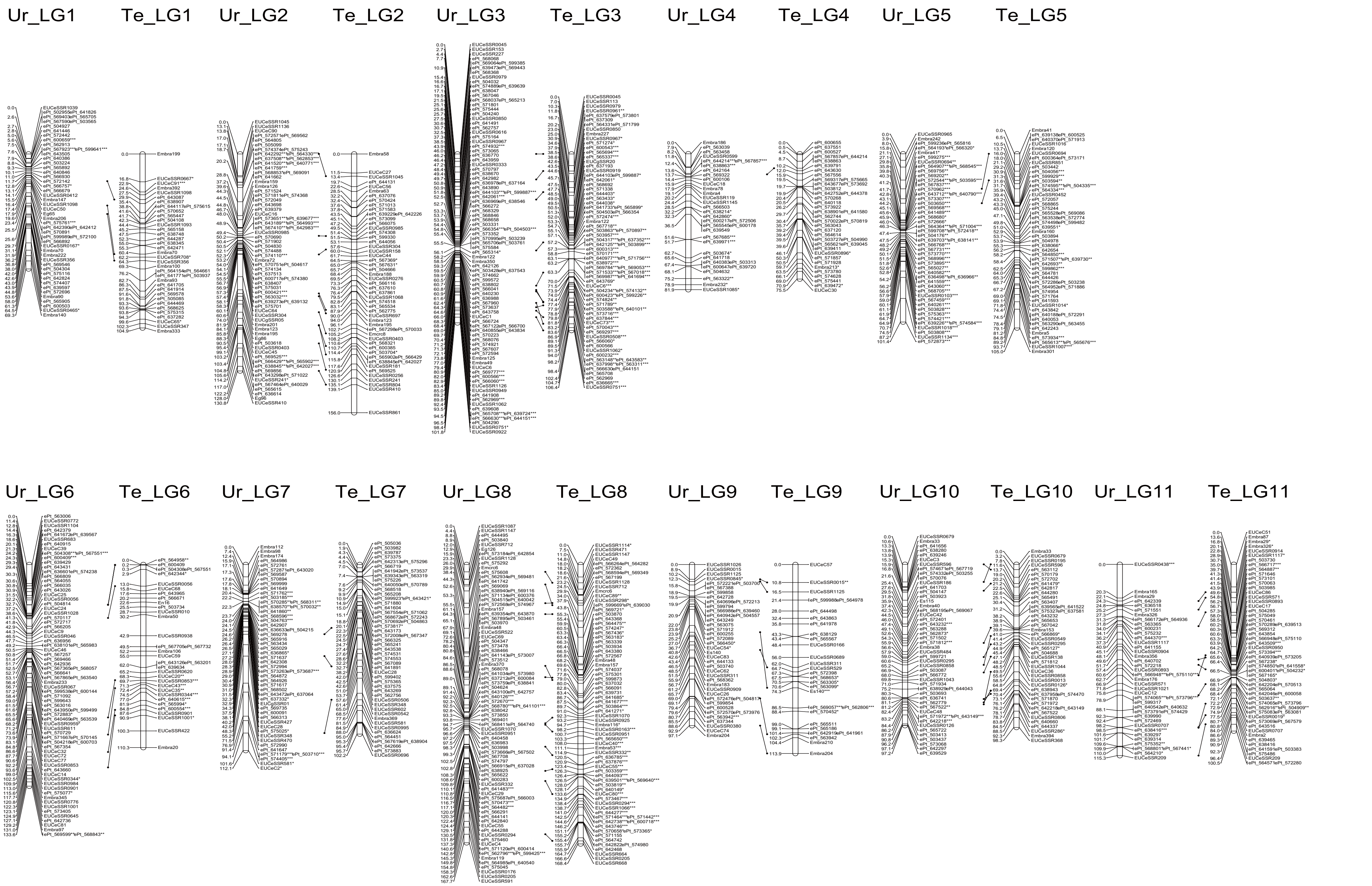
Ur_LG1
Te_LG1
Ur_LG2
Te_LG2
Ur_LG3
Te_LG3
Ur_LG4
Te_LG4
Ur_LG5
Te_LG5
Ur_LG6
Te_LG6
Ur_LG7
Te_LG7
Ur_LG8
Te_LG8
Ur_LG9
Te_LG9
Ur_LG10
Te_LG10
Ur_LG11
Te_LG11

S1 Fig. Genetic maps of *Eucalyptus urophylla* (Ur) and *E. tereticornis* (Te). Linkage groups (LGs) are designated according to Brondani et al. [34]. Accumulated map distances (cM) and locus names are presented to the left and right of each LG, respectively. Common markers in both maps are connected by a line. Marker segregation distortions are marked with * ($P \leq 0.05$), ** ($P \leq 0.01$), and *** ($P \leq 0.001$).
