# Supplementary material for: Comparative Genomics Analyses Reveal Extensive Chromosome Colinearity and Novel Quantitative Trait Loci in Eucalyptus
Source: PLoS One. 2015 Dec 22;10(12):e0145144. doi: 10.1371/journal.pone.0145144 (PMC4687840; doi:10.1371/journal.pone.0145144)
Supplement: S2 Fig — Each linkage group is the same as shown in S1 Fig A black dashed line indicates the LOD threshold (usually being 3.4 and 3.3 or 3.2 in a few cases) used for declaring a QTL in software MapQTL 6.0 [44]. (PDF) [file pone.0145144.s002.pdf]

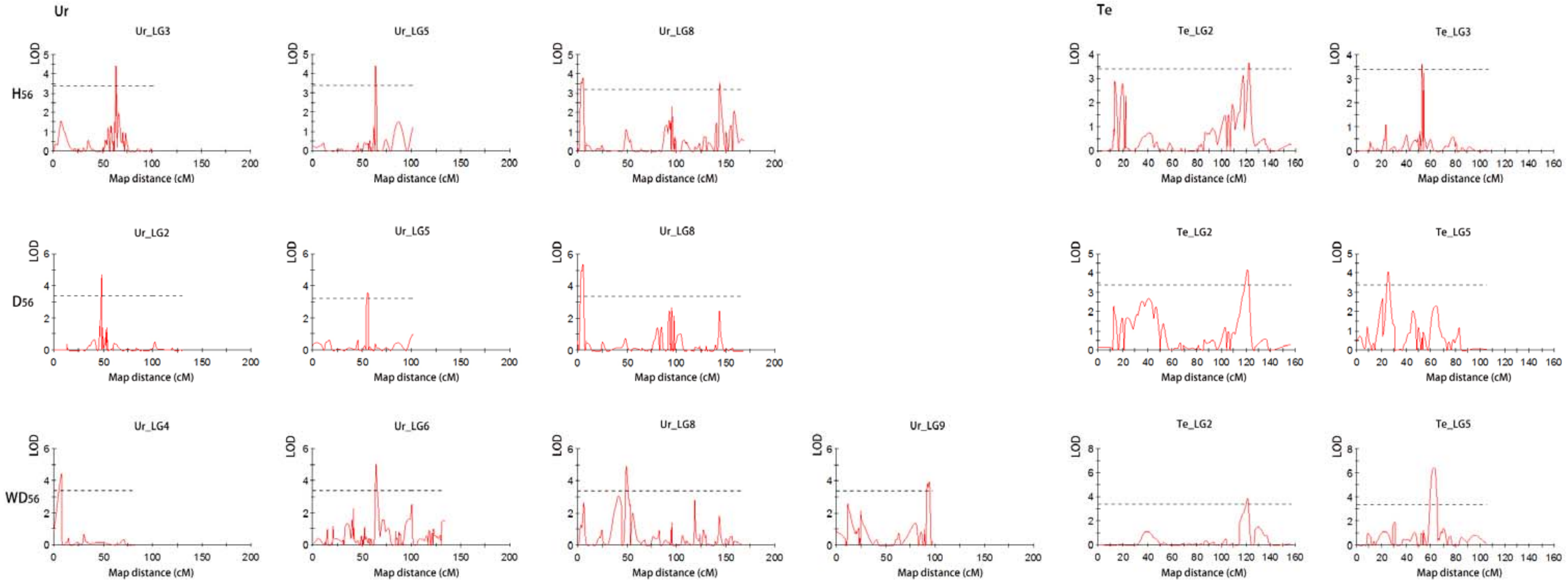

S2 Fig. QTL analysis for 56-month-old height (H_{56}), diameter at breast height (D_{56}), and wood density (WD_{56}) in *Eucalyptus urophylla* (Ur) and *E. tereticornis* (Te). Each linkage group is the same as shown in S1 Fig. A black dashed line indicates the LOD threshold (usually being 3.4 and 3.3 or 3.2 in a few cases) used for declaring a QTL in software MapQTL 6.0 [44].
