# Supplementary material for: Comparative Genomics Analyses Reveal Extensive Chromosome Colinearity and Novel Quantitative Trait Loci in Eucalyptus
Source: PLoS One. 2015 Dec 22;10(12):e0145144. doi: 10.1371/journal.pone.0145144 (PMC4687840; doi:10.1371/journal.pone.0145144)
Supplement: S1 Table — (DOC) [file pone.0145144.s003.doc]

**S1 Table. Means (standard deviations, SD) and ranges for height (*H*) at 10, 23, 32, 44, and 56 months of age (*H*_10_, *H*_23_, *H*_32_, *H*_44_, and *H*_56_, respectively), diameter at breast height (*D*) at 23, 32, 44, and 56 months (*D*_23_, *D*_32_, *D*_44_, and *D*_56_, respectively), and 56-month-old wood density (*WD*_56_) measured in the *E. urophylla* × *E. tereticornis* mapping population.**

| **Trait** | **Mean (SD)** | **Minimum** | **Maximum** |
| --- | --- | --- | --- |
| *H*_10_ (m) | 3.54 (0.59) | 1.68 | 4.60 |
| *H*_23_ (m) | 9.23 (1.17) | 3.82 | 11.50 |
| *H*_32_ (m) | 10.54 (1.38) | 4.33 | 12.28 |
| *H*_44_ (m) | 15.53 (2.94) | 5.75 | 20.50 |
| *H*_56_ (m) | 17.73 (3.57) | 6.53 | 22.63 |
| *D*_23_ (cm) | 7.70 (1.09) | 3.60 | 9.82 |
| *D*_32_ (cm) | 9.23 (1.67) | 3.87 | 12.30 |
| *D*_44_ (cm) | 11.54 (2.56) | 4.47 | 17.38 |
| *D*_56_ (cm) | 12.28 (3.16) | 5.13 | 19.43 |
| *WD*_56_ (%) | 22.20 (4.02) | 13.46 | 31.29 |
