# Supplementary material for: Comparative Genomics Analyses Reveal Extensive Chromosome Colinearity and Novel Quantitative Trait Loci in Eucalyptus
Source: PLoS One. 2015 Dec 22;10(12):e0145144. doi: 10.1371/journal.pone.0145144 (PMC4687840; doi:10.1371/journal.pone.0145144)
Supplement: S2 Table — (DOC) [file pone.0145144.s004.doc]

**S2 Table. Mean squares from ANOVA for growth and wood density of the *E. urophylla* × *E. tereticornis*** mapping population.

| **Source of variation** | **d.f.** | ***H*_10_** | ***H*_23_** | ***H*_32_** | ***H*_44_** | ***H*_56_** | ***D*_23_** | ***D*_32_** | ***D*_44_** | ***D*_56_** | ***WD*_56_** |
| --- | --- | --- | --- | --- | --- | --- | --- | --- | --- | --- | --- |
| Replicate | 5 | 3.97^a^ | 17.19^a^ | 5.44^b^ | 2.50^c^ | 9.40^c^ | 10.68^a^ | 8.20^b^ | 10.65^c^ | 9.45^c^ | 73.60^a^ |
| Full-sib | 131^d^ | 1.70^a^ | 6.18^a^ | 9.04^a^ | 32.61^a^ | 35.41^a^ | 5.46^a^ | 12.97^a^ | 26.68^a^ | 30.69^a^ | 51.91^a^ |
| Error | - ^e^ | 0.43 | 1.21 | 1.75 | 5.94 | 6.38 | 1.37 | 2.38 | 4.93 | 6.01 | 10.40 |

Trait abbreviations are as illustrated in S1 Table.

^a^ *P* < 0.001.

^b^ 0.001 ≤ *P* < 0.01.

^c^ Not significant at 0.05 level.

^d^ The d.f. value for full-sib in trait *WD*_56_ is 124.

^e^ The d.f. value for error varies with trait from 293 in *WD*_56_ to 516 in *H*_10_.
