# Supplementary material for: Comparative Genomics Analyses Reveal Extensive Chromosome Colinearity and Novel Quantitative Trait Loci in Eucalyptus
Source: PLoS One. 2015 Dec 22;10(12):e0145144. doi: 10.1371/journal.pone.0145144 (PMC4687840; doi:10.1371/journal.pone.0145144)
Supplement: S3 Table — (DOC) [file pone.0145144.s005.doc]

**S3 Table. Pearson correlation coefficients between traits of the mapping population.**

| **Trait** | ***H*_23_** | ***H*_32_** | ***H*_44_** | ***H*_56_** | ***D*_23_** | ***D*_32_** | ***D*_44_** | ***D*_56_** | ***WD*_56_** |
| --- | --- | --- | --- | --- | --- | --- | --- | --- | --- |
| *H*_10_ | 0.68^a^ | 0.57^a^ | 0.55^a^ | 0.52^a^ | 0.72^a^ | 0.67^a^ | 0.54^a^ | 0.45^a^ | 0.43^a^ |
| *H*_23_ |  | 0.75^a^ | 0.60^a^ | 0.61^a^ | 0.69^a^ | 0.66^a^ | 0.50^a^ | 0.43^a^ | 0.37^a^ |
| *H*_32_ |  |  | 0.71^a^ | 0.71^a^ | 0.64^a^ | 0.72^a^ | 0.59^a^ | 0.55^a^ | 0.37^a^ |
| *H*_44_ |  |  |  | 0.94^a^ | 0.65^a^ | 0.75^a^ | 0.81^a^ | 0.81^a^ | 0.57^a^ |
| *H*_56_ |  |  |  |  | 0.68^a^ | 0.79^a^ | 0.85^a^ | 0.85^a^ | 0.61^a^ |
| *D*_23_ |  |  |  |  |  | 0.93^a^ | 0.80^a^ | 0.76^a^ | 0.43^a^ |
| *D*_32_ |  |  |  |  |  |  | 0.93^a^ | 0.91^a^ | 0.51^a^ |
| *D*_44_ |  |  |  |  |  |  |  | 0.99^a^ | 0.59^a^ |
| *D*_56_ |  |  |  |  |  |  |  |  | 0.59^a^ |

Trait abbreviations are as illustrated in S1 Table. Phenotypic correlation between height and diameter of the same month (underlined) appears to increase with age.

^a^ *P* < 0.001.
