# Supplementary material for: Comparative Genomics Analyses Reveal Extensive Chromosome Colinearity and Novel Quantitative Trait Loci in Eucalyptus
Source: PLoS One. 2015 Dec 22;10(12):e0145144. doi: 10.1371/journal.pone.0145144 (PMC4687840; doi:10.1371/journal.pone.0145144)
Supplement: S4 Table — (DOC) [file pone.0145144.s006.doc]

**S4 Table. Number of diversity arrays technology (DArT), genomic simple sequence repeats (gSSR), expressed sequence tag-derived SSR (EST-SSR), and EST-derived cleaved amplified polymorphic sequence (CAPS) markers segregating in the *E. urophylla* × *E. tereticornis* mapping population.**

| **Marker type** | **1:1** | | | **3:1** | **1:2:1** | **1:1:1:1** | **Sub-total** |
| --- | --- | --- | --- | --- | --- | --- | --- |
|  | **Female (Ur)** | **Male (Te)** | **Sub-total** |  |  |  |  |
| DArT | 494 | 395 | 889 | 298 | -^a^ | -^a^ | 1,187 |
| gSSR | 60 | 54 | 114 | 0 | 2 | 65 | 181 |
| EST-SSR | 34 | 67 | 101 | 0 | 6 | 127 | 234 |
| EST-CAPS | 38 | 38 | 76 | 0 | 0 | 15 | 91 |
| Sub-total | 626 | 554 | 1,180 | 298 | 8 | 207 |  |
| Total |  |  |  |  |  |  | 1,693 |

^a^ Inapplicable to the marker type.
