# Supplementary material for: Comparative Genomics Analyses Reveal Extensive Chromosome Colinearity and Novel Quantitative Trait Loci in Eucalyptus
Source: PLoS One. 2015 Dec 22;10(12):e0145144. doi: 10.1371/journal.pone.0145144 (PMC4687840; doi:10.1371/journal.pone.0145144)
Supplement: S5 Table — (DOC) [file pone.0145144.s007.doc]

**S5 Table. Number of markers showing segregation distortion at various significant levels in the *E. urophylla* × *E. tereticornis* mapping population.**

| **Marker type** | **1:1 (P_1_/P_2_)** | | |  | **3:1 in DArT or 1:2:1 in SSR** | | |  | **1:1:1:1** | | |  | **Sub-total** | | |
| --- | --- | --- | --- | --- | --- | --- | --- | --- | --- | --- | --- | --- | --- | --- | --- |
|  | ***P <* 0.05** | ***P* < 0.01** | ***P* < 0.001** |  | ***P <* 0.05** | ***P* < 0.01** | ***P* < 0.001** |  | ***P <* 0.05** | ***P* < 0.01** | ***P* < 0.001** |  | ***P <* 0.05** | ***P* < 0.01** | ***P* < 0.001** |
| DArT | 224 (89/135) | 157 (65/92) | 89 (50/39) |  | 77 | 35 | 19 |  | -^a^ | -^a^ | -^a^ |  | 301 | 192 | 108 |
| gSSR | 9 (2/7) | 0 | 0 |  | 1 | 0 | 0 |  | 11 | 0 | 0 |  | 21 | 0 | 0 |
| EST-SSR | 14 (4/10) | 4 (1/3) | 4 (1/3) |  | 5 | 4 | 4 |  | 69 | 58 | 53 |  | 88 | 66 | 61 |
| EST-CAPS | 17 (3/14) | 12 (0/12) | 7 (0/7) |  | -^a^ | -^a^ | -^a^ |  | 6 | 4 | 2 |  | 23 | 16 | 9 |
| Sub-total | 264 (98/166) | 173 (66/107) | 100 (51/49) |  | 83 | 39 | 23 |  | 86 | 62 | 55 |  |  |  |  |
| Total |  |  |  |  |  |  |  |  |  |  |  |  | 433 (25.6%) | 274 (16.2%) | 178 (10.5%) |

Marker type abbreviations are as illustrated in S4 Table.

^a^ Inapplicable to the marker type.
