# Supplementary material for: Comparative Genomics Analyses Reveal Extensive Chromosome Colinearity and Novel Quantitative Trait Loci in Eucalyptus
Source: PLoS One. 2015 Dec 22;10(12):e0145144. doi: 10.1371/journal.pone.0145144 (PMC4687840; doi:10.1371/journal.pone.0145144)
Supplement: S8 Table — (DOC) [file pone.0145144.s010.doc]

**S8 Table. Sequence identity (%) between DArT markers clustered (with five or more markers within 0.01 cM) on the genetic maps of *E. urophylla* (Ur) and *E. tereticornis* (Te).**

| **LG and marker  (map position, cM)** | **Sequence identity (%)** | | | | | |
| --- | --- | --- | --- | --- | --- | --- |
| **Ur_LG1** | **ePt_ 503565** | **ePt_ 565705** | **ePt_ 567590** | **ePt_ 569403** |  |  |
| ePt_641826 (2.605) | 36.3 | 35.9 | 31.9 | 89.9 |  |  |
| ePt_503565 (2.614) |  | 88.8 | 96.2 | 34.6 |  |  |
| ePt_565705 (2.614) |  |  | 90.1 | 38.5 |  |  |
| ePt_567590 (2.614) |  |  |  | 37.4 |  |  |
| ePt_569403 (2.614) |  |  |  |  |  |  |
| **Ur_LG2** | **ePt_ 564330** | **ePt_ 637508** | **ePt_ 642292** | **ePt_ 640771** | **ePt_ 641520** | **ePt_ 641759** |
| ePt_562853 (20.195) | 36.0 | 35.5 | 36.4 | 96.4 | 36.5 | 95.8 |
| ePt_564330 (20.195) |  | 93.5 | 95.9 | 35.8 | 93.7 | 37.6 |
| ePt_637508 (20.195) |  |  | 97.0 | 36.9 | 93.9 | 36.7 |
| ePt_642292 (20.195) |  |  |  | 34.7 | 94.8 | 37.6 |
| ePt_640771 (20.201) |  |  |  |  | 37.1 | 96.9 |
| ePt_641520 (20.201) |  |  |  |  |  | 40.2 |
| ePt_641759 (20.201) |  |  |  |  |  |  |
| **Ur_LG3** | **ePt_ 565706** | **ePt_ 570995** | **ePt_ 575584** | **ePt_ 503761** |  |  |
| ePt_503239 (55.527) | 38.1 | 41.9 | 40.1 | NA |  |  |
| ePt_565706 (55.527) |  | 90.7 | 93.8 | NA |  |  |
| ePt_570995 (55.527) |  |  | 93.1 | NA |  |  |
| ePt_575584 (55.527) |  |  |  | NA |  |  |
| ePt_503761 (55.527) |  |  |  |  |  |  |
| **Ur_LG4** | **ePt_ 600178** | **ePt_ 639549** | **ePt_ 572506** | **ePt_ 600217** |  |  |
| ePt_565545 (36.513) | 93.6 | 93.2 | NA | NA |  |  |
| ePt_600178 (36.513) |  | 97.7 | NA | NA |  |  |
| ePt_639549 (36.513) |  |  | NA | NA |  |  |
| ePt_572506 (36.513) |  |  |  | NA |  |  |
| ePt_600217 (36.513) |  |  |  |  |  |  |
| **Ur_LG7** | **ePt_ 570032** | **ePt_ 570285** | **ePt_ 638570** | **ePt_ 641860** |  |  |
| ePt_568311 (22.316) | 94.6 | 94.2 | 34.7 | NA |  |  |
| ePt_570032 (22.316) |  | 99.0 | 35.8 | NA |  |  |
| ePt_570285 (22.316) |  |  | 37.8 | NA |  |  |
| ePt_638570 (22.316) |  |  |  | NA |  |  |
| ePt_641860 (22.324) |  |  |  |  |  |  |
| **Ur_LG8** | **ePt_ 573759** | **ePt_ 600084** | **ePt_ 638841** | **ePt_ 641034** | **ePt_ 637212** |  |
| ePt_573980 (88.979) | 39.8 | 86.6 | 84.6 | 39.6 | NA |  |
| ePt_573759 (88.979) |  | 42.0 | 40.1 | 93.7 | NA |  |
| ePt_600084 (88.979) |  |  | 97.5 | 38.1 | NA |  |
| ePt_638841 (88.979) |  |  |  | 38.1 | NA |  |
| ePt_641034 (88.979) |  |  |  |  | NA |  |
| ePt_637212 (88.979) |  |  |  |  |  |  |
| **Te_LG3** | **ePt_ 569053** | **ePt_ 569987** | **ePt_ 571533** | **ePt_ 569784** | **ePt_ 641694** |  |
| ePt_567018 (63.250) | 99.0 | 36.3 | 91.2 | NA | 98.9 |  |
| ePt_569053 (63.250) |  | 33.3 | 99.5 | NA | 98.7 |  |
| ePt_569987 (63.250) |  |  | 33.2 | NA | 35.9 |  |
| ePt_571533 (63.250) |  |  |  | NA | 98.9 |  |
| ePt_569784 (63.250) |  |  |  |  | NA |  |
| ePt_641694 (63.255) |  |  |  |  |  |  |

NA, not available due to absence in sequence of one or two of the DArT markers.
