# Supplementary material for: Comparative Genomics Analyses Reveal Extensive Chromosome Colinearity and Novel Quantitative Trait Loci in Eucalyptus
Source: PLoS One. 2015 Dec 22;10(12):e0145144. doi: 10.1371/journal.pone.0145144 (PMC4687840; doi:10.1371/journal.pone.0145144)
Supplement: S9 Table — (DOC) [file pone.0145144.s011.doc]

**S9 Table. The best linear unbiased prediction (BLUP) trait values for the clonal full-sibs of the mapping population.**

| **Full- sib** | ***H*_10_** (m) [3.54]^a^ | ***H*_23_** (m) [9.23]^a^ | ***H*_32_** (m) [10.58]^a^ | ***H*_44_** (m) [15.84]^a^ | ***H*_56_** (m) [18.10]^a^ | ***D*_23_** (cm) [7.23]^a^ | ***D*_32_** (cm) [9.29]^a^ | ***D*_44_** (cm) [11.79]^a^ | ***D*_56_** (cm) [12.56]^a^ | ***WD*_56_** (%) [22.37]^a^ |
| --- | --- | --- | --- | --- | --- | --- | --- | --- | --- | --- |
| 1 | 0.42 | 0.65 | 0.80 | 3.39 | 3.41 | 0.51 | 1.36 | 2.43 | 3.67 | 2.01 |
| 2 | -0.41 | -1.60 | -1.59 | -2.70 | -5.05 | -0.56 | -1.74 | -1.92 | -3.57 | -2.27 |
| 3 | 0.39 | 0.86 | 0.82 | 3.62 | 3.82 | 0.64 | 1.17 | 1.87 | 2.21 | 5.23 |
| 4 | -0.31 | -1.02 | -0.74 | -3.11 | -3.78 | -1.02 | -1.82 | -2.95 | -3.27 | 0.00 |
| 5 | -0.15 | -0.03 | 0.13 | -2.12 | -2.31 | -0.53 | -0.88 | -1.84 | -2.78 | -0.59 |
| 6 | 0.19 | 0.84 | 0.90 | 1.66 | 2.98 | 0.71 | 1.30 | 1.10 | 1.75 | 1.48 |
| 7 | 0.09 | 0.83 | 0.39 | 1.17 | -1.04 | -0.22 | -0.29 | -0.87 | -1.20 | 2.46 |
| 8 | -0.64 | -0.84 | -0.89 | -0.87 | -1.37 | -1.56 | -2.34 | -1.26 | -2.16 | -0.68 |
| 9 | 0.27 | 0.56 | 0.46 | 0.33 | -0.69 | 0.45 | 0.48 | 0.05 | -0.98 | 3.28 |
| 10 | 0.33 | 0.64 | 0.63 | 1.39 | 1.85 | 0.46 | 0.81 | 1.44 | 1.52 | 2.62 |
| 11 | -0.19 | 0.43 | 0.49 | -0.07 | -0.96 | 0.08 | 0.47 | 0.08 | -1.27 | -2.66 |
| 12 | -0.30 | -0.01 | 0.02 | -1.27 | -2.35 | -0.35 | -1.38 | -2.12 | -3.23 | -1.84 |
| 13 | 0.33 | 0.46 | 0.69 | 1.19 | 0.55 | 0.26 | 0.54 | 0.34 | -0.27 | 3.58 |
| 14 | -1.45 | -4.44 | -5.20 | -8.55 | -9.26 | -3.18 | -4.57 | -6.15 | -5.77 | -5.89 |
| 15 | -0.16 | 0.79 | 0.97 | 1.04 | 1.36 | 1.23 | 1.91 | 3.12 | 3.48 | 1.36 |
| 16 | -0.33 | -0.68 | 0.02 | -2.10 | -3.31 | -1.01 | -1.72 | -3.45 | -4.24 | -5.54 |
| 17 | 0.19 | 0.43 | 0.67 | 1.65 | 1.26 | 0.65 | 1.23 | 2.28 | 1.57 | 1.53 |
| 18 | 0.18 | 0.46 | 0.41 | 0.93 | 1.11 | -0.10 | 0.08 | -0.24 | 0.02 | 3.99 |
| 19 | 0.35 | 0.93 | 1.15 | 3.03 | 3.73 | 0.47 | 0.95 | 1.85 | 2.84 | 4.95 |
| 20 | 0.31 | 0.62 | 1.02 | 2.02 | 2.34 | 0.99 | 2.11 | 3.43 | 4.61 | -0.42 |
| 21 | -0.97 | -1.05 | -0.42 | -2.76 | -3.76 | -1.45 | -1.85 | -2.82 | -2.52 | -2.76 |
| 22 | -0.24 | 0.41 | 0.77 | 1.51 | 1.92 | 0.23 | 0.64 | 0.70 | 1.15 | -0.45 |
| 23 | 0.53 | 0.67 | 0.91 | 2.32 | 3.22 | 0.30 | 0.61 | 0.75 | 1.11 | 3.32 |
| 24 | 0.58 | 0.41 | 0.74 | 1.05 | 0.31 | -0.03 | -0.21 | -0.98 | -1.48 | -1.94 |
| 25 | -0.83 | 0.00 | -0.65 | 1.23 | 1.65 | 0.41 | 0.23 | 1.30 | 1.47 | -4.46 |
| 26 | -0.51 | -0.97 | -0.60 | -2.45 | -4.04 | -0.35 | -0.48 | -1.56 | -2.38 | -4.75 |
| 27 | -0.23 | -0.08 | 0.59 | 0.86 | 0.31 | 0.41 | 0.58 | -0.10 | -0.64 | -5.43 |
| 28 | -0.15 | 0.35 | 0.04 | 0.44 | 0.91 | -0.04 | -0.06 | -0.45 | 0.54 | 0.76 |
| 29 | -0.28 | -0.31 | 0.13 | -1.63 | -0.64 | -0.13 | -0.44 | -1.74 | -1.30 | -3.40 |
| 30 | 0.28 | 0.50 | 0.20 | 0.96 | 2.53 | 0.56 | 0.81 | 0.57 | 1.44 | -0.04 |
| 31 | -0.65 | -0.57 | -1.79 | -2.13 | -3.11 | -0.92 | -1.38 | -1.90 | -2.13 | -3.55 |
| 32 | -0.34 | 0.16 | -0.40 | -2.65 | -3.51 | -0.70 | -1.07 | -2.13 | -3.86 | -6.51 |
| 33 | 0.40 | 0.46 | 0.36 | 1.27 | 1.55 | 0.10 | 0.34 | 0.15 | 0.17 | 1.20 |
| 34 | -0.84 | -0.52 | -0.34 | -0.83 | -1.24 | -0.92 | -1.18 | -1.92 | -2.37 | 0.83 |
| 35 | 0.20 | 0.68 | 0.88 | 1.75 | 3.22 | 1.14 | 1.43 | 2.28 | 3.07 | 0.33 |
| 36 | 0.15 | 0.69 | 0.93 | 1.88 | 1.72 | 0.31 | 0.47 | 0.62 | 0.98 | 2.46 |
| 37 | 0.01 | -0.03 | 0.30 | -0.78 | -0.88 | -0.79 | -1.26 | -1.90 | -2.22 | -1.21 |
| 38 | -0.27 | -0.79 | -1.07 | -3.59 | -4.46 | -0.89 | -1.50 | -3.01 | -3.78 | -5.18 |
| 39 | -1.37 | -3.53 | -4.53 | -4.05 | -5.90 | -2.05 | -4.02 | -1.98 | -2.77 | -3.07 |
| 40 | -0.61 | -0.20 | -0.88 | -2.19 | -2.54 | -1.03 | -1.47 | -2.56 | -3.23 | -2.31 |
| 41 | -0.53 | -0.43 | -0.22 | 0.91 | 1.19 | -0.04 | -0.07 | 1.33 | 0.50 | -1.46 |
| 42 | 0.21 | 0.27 | -0.39 | 0.02 | -0.67 | 0.73 | 0.89 | 0.25 | -0.48 | 2.53 |
| 43 | -0.53 | -2.75 | -3.39 | -3.45 | 0.00 | -1.76 | -2.63 | -2.71 | 0.00 | 0.00 |
| 44 | -0.49 | -0.76 | -0.74 | -2.66 | -3.64 | 0.22 | 0.10 | -1.35 | -1.82 | -3.63 |
| 45 | 0.36 | 0.78 | 0.63 | 1.40 | 1.36 | -0.10 | 1.30 | 1.73 | 1.54 | -0.15 |
| 46 | 0.18 | 0.22 | 0.69 | 1.97 | 2.94 | 0.66 | 1.26 | 1.57 | 3.05 | 3.62 |
| 47 | 0.21 | 0.46 | 0.28 | -0.66 | -0.75 | 0.60 | 0.54 | -0.60 | -1.21 | -2.57 |
| 48 | 0.07 | 0.34 | 0.16 | 0.63 | -0.09 | 0.18 | 0.17 | 0.24 | 0.35 | -2.80 |
| 49 | 0.04 | 0.55 | 0.86 | 1.84 | 2.64 | 0.40 | 1.03 | 1.59 | 2.27 | -0.42 |
| 50 | 0.55 | 0.55 | 0.57 | 1.72 | 1.68 | 0.43 | 0.81 | 1.01 | 0.25 | 4.91 |
| 51 | -0.47 | -0.12 | 0.60 | -1.43 | -2.52 | -0.64 | -0.88 | -1.97 | -2.30 | -3.40 |
| 52 | -0.29 | 0.58 | 0.39 | 2.23 | 3.15 | 0.51 | 1.18 | 1.72 | 3.11 | 3.31 |
| 53 | 0.16 | 0.02 | 0.27 | 2.99 | 2.98 | 0.66 | 1.44 | 2.04 | 1.95 | 2.67 |
| 54 | -0.64 | -1.05 | -0.99 | -2.59 | -3.29 | -1.47 | -2.19 | -3.10 | -3.74 | -4.30 |
| 55 | 0.15 | 0.68 | 1.26 | 3.23 | 4.05 | 0.88 | 1.77 | 3.79 | 4.99 | 5.45 |
| 56 | -0.32 | 0.19 | 0.00 | -1.73 | -0.78 | -0.77 | -1.03 | -1.98 | -1.41 | 1.51 |
| 57 | 0.61 | 0.73 | 0.95 | 2.91 | 2.84 | 1.11 | 2.16 | 4.03 | 4.98 | 1.64 |
| 58 | 0.48 | 0.44 | 0.77 | 2.93 | 2.06 | 0.49 | 0.97 | 1.78 | 1.79 | 4.46 |
| 59 | 0.25 | -0.05 | 0.38 | 0.07 | -0.33 | 0.51 | 0.53 | 0.24 | -0.98 | -0.80 |
| 60 | -0.26 | -0.24 | 0.36 | 1.04 | 1.21 | -0.25 | -0.19 | 0.12 | 0.35 | -1.32 |
| 61 | -0.05 | 0.71 | 0.95 | 2.35 | 1.24 | 0.14 | 0.56 | 1.00 | 1.44 | 0.56 |
| 62 | -1.13 | -2.81 | -3.09 | -5.61 | -6.40 | -1.63 | -2.94 | -3.45 | -3.68 | 0.00 |
| 63 | 0.31 | 0.44 | 0.64 | -0.03 | 0.17 | -0.22 | -0.70 | -0.69 | -1.06 | 3.32 |
| 64 | 0.17 | -0.14 | -0.90 | -0.97 | -0.73 | 0.62 | 0.75 | 0.06 | 2.49 | -0.19 |
| 65 | 0.12 | 0.05 | -0.40 | -0.59 | 0.04 | -0.43 | -0.25 | -0.57 | -0.43 | 1.46 |
| 66 | -0.52 | -0.82 | -0.85 | -3.47 | -4.10 | -0.52 | -1.34 | -2.68 | -3.44 | -6.33 |
| 67 | -0.41 | -1.79 | -1.61 | -2.10 | -2.17 | -1.12 | -1.50 | -1.78 | -2.26 | 0.58 |
| 68 | -0.80 | -0.54 | -0.61 | -1.51 | -1.69 | -0.43 | -0.56 | -1.63 | -1.77 | -1.57 |
| 69 | 0.23 | 0.64 | 0.82 | 1.88 | 2.24 | 0.93 | 1.38 | 1.96 | 2.09 | 1.17 |
| 70 | -0.07 | 0.26 | 0.62 | 1.29 | 1.64 | 0.63 | 0.91 | 0.77 | 1.46 | -0.34 |
| 71 | -0.73 | -0.31 | 0.19 | 1.98 | 2.16 | -0.19 | -0.15 | 1.31 | 1.31 | -1.08 |
| 72 | 0.19 | 0.50 | 1.09 | 1.59 | 3.33 | 1.67 | 2.57 | 3.89 | 5.01 | 1.80 |
| 73 | 0.68 | 0.95 | 0.72 | 1.81 | 2.38 | 1.41 | 2.02 | 2.81 | 3.53 | -0.07 |
| 74 | 0.25 | 0.09 | 0.50 | 0.46 | 0.53 | 0.63 | 0.64 | 0.90 | 0.58 | -0.89 |
| 75 | -0.19 | -0.83 | 0.00 | 0.17 | 0.05 | -0.11 | -0.15 | -0.76 | -0.84 | -2.31 |
| 76 | 0.28 | 0.01 | 0.59 | 1.00 | -0.30 | 0.77 | 0.98 | 1.45 | -0.10 | 0.06 |
| 77 | 0.71 | 0.75 | 0.66 | 2.57 | 3.71 | 0.76 | 1.08 | 3.33 | 3.38 | 6.13 |
| 78 | -0.81 | -1.99 | -2.61 | -4.26 | 0.00 | -1.98 | -3.13 | -3.63 | 0.00 | 0.00 |
| 79 | -0.02 | 0.17 | -2.64 | -2.41 | -3.24 | -0.72 | -3.14 | -2.68 | -3.38 | -3.51 |
| 80 | -0.41 | -0.08 | 0.31 | -2.10 | -2.69 | -0.74 | -1.14 | -2.38 | -2.67 | -5.80 |
| 81 | 0.33 | 0.65 | 0.71 | 2.67 | 2.75 | 0.75 | 0.91 | 0.92 | 1.58 | 1.27 |
| 82 | 0.05 | 0.15 | 0.22 | -0.97 | -1.50 | -0.16 | -0.38 | -1.42 | -2.04 | 0.17 |
| 83 | -0.25 | -1.41 | -0.47 | -3.73 | -4.83 | -0.17 | -0.23 | -1.85 | -1.92 | -3.32 |
| 84 | 0.19 | 0.64 | 0.45 | 1.29 | 1.02 | 0.65 | 1.12 | 1.81 | 1.75 | 0.09 |
| 85 | 0.37 | 0.67 | 0.55 | -0.02 | -0.79 | -0.03 | -0.04 | -0.32 | -1.79 | 0.26 |
| 86 | 0.41 | 0.67 | 0.17 | -0.33 | -0.55 | 0.16 | 0.20 | -0.92 | -1.32 | -1.29 |
| 87 | -0.11 | 0.51 | 0.34 | -0.44 | -1.05 | 0.31 | 0.37 | -0.16 | -0.54 | -3.44 |
| 88 | 0.31 | 0.64 | 0.72 | 2.87 | 2.77 | 1.00 | 1.56 | 2.66 | 3.19 | 4.18 |
| 89 | 0.42 | 0.73 | 0.57 | 1.32 | 1.84 | 0.21 | 0.46 | 0.75 | 0.44 | 3.06 |
| 90 | 0.40 | 0.62 | 0.88 | 2.44 | 2.92 | 0.46 | 1.62 | 2.60 | 3.05 | 6.05 |
| 91 | -0.35 | -1.27 | -1.89 | -5.45 | -6.54 | -1.18 | -2.39 | -3.92 | -3.72 | 0.00 |
| 92 | -0.18 | -1.18 | 0.09 | 0.45 | 0.51 | -0.53 | 0.20 | 0.95 | 0.27 | -0.87 |
| 93 | -0.48 | -1.40 | -1.43 | -3.49 | -4.33 | -1.05 | -1.78 | -1.32 | -0.96 | 1.66 |
| 94 | 0.60 | 0.84 | 1.09 | 0.83 | 0.63 | 0.57 | 0.95 | 0.89 | 0.90 | 3.64 |
| 95 | 0.21 | -0.15 | -0.32 | -1.75 | -1.90 | -0.27 | 0.13 | 0.51 | 0.54 | -4.87 |
| 96 | -0.31 | -0.20 | -0.02 | -1.60 | -1.64 | -0.34 | -0.52 | -1.35 | -1.64 | 0.70 |
| 97 | 0.49 | 0.66 | 0.61 | 1.36 | 2.86 | 0.26 | 0.86 | 1.77 | 2.57 | 3.23 |
| 98 | 0.16 | 0.85 | 0.68 | 1.61 | 2.68 | 0.14 | 0.48 | 0.45 | 1.08 | 2.85 |
| 99 | 0.22 | 0.37 | 0.33 | 0.25 | 0.21 | 0.47 | 0.60 | 0.08 | -0.04 | -4.53 |
| 100 | 0.40 | 0.39 | 0.35 | 1.57 | 1.62 | 0.19 | 0.44 | 0.68 | 1.13 | 2.54 |
| 101 | 0.22 | 0.17 | 0.20 | 1.17 | 1.57 | 0.14 | -0.13 | 0.00 | -0.06 | 6.89 |
| 102 | -0.37 | -1.07 | -0.60 | -3.62 | -5.11 | 0.63 | -0.13 | -1.40 | -3.05 | -3.70 |
| 103 | 0.16 | 0.20 | -2.45 | 0.51 | 0.73 | 0.54 | -1.45 | 0.78 | 0.79 | 0.83 |
| 104 | 0.29 | 0.21 | 0.27 | 0.35 | 1.85 | -0.01 | 0.37 | 0.72 | 1.42 | 3.80 |
| 105 | -0.26 | -1.49 | -1.38 | -2.47 | 0.00 | -0.75 | -1.65 | -1.52 | 0.00 | 0.00 |
| 106 | 0.54 | 0.83 | 1.23 | 3.12 | 2.95 | 1.13 | 2.43 | 4.76 | 5.53 | 3.19 |
| 107 | 0.57 | 0.67 | 0.91 | 1.86 | 2.83 | 0.73 | 1.03 | 1.69 | 1.75 | -1.62 |
| 108 | 0.00 | 0.06 | 0.45 | -1.20 | -0.98 | 0.05 | 0.16 | -0.45 | -1.42 | -3.48 |
| 109 | -0.50 | -2.11 | -2.25 | -5.40 | -7.20 | -1.49 | -2.58 | -3.75 | -4.60 | 0.00 |
| 110 | 0.26 | 1.09 | 0.92 | 3.29 | 4.05 | 0.81 | 1.66 | 3.27 | 4.07 | 2.15 |
| 111 | 0.25 | 0.61 | 0.62 | 2.40 | 2.43 | 0.63 | 1.31 | 2.27 | 3.69 | 0.11 |
| 112 | 0.34 | 0.74 | 0.48 | 1.09 | 3.02 | 0.94 | 1.36 | 1.92 | 2.84 | 0.10 |
| 113 | 0.12 | -0.80 | -0.27 | -1.51 | 0.00 | 0.16 | -0.34 | -0.58 | 0.00 | 0.00 |
| 114 | 0.13 | 0.95 | -0.58 | 1.67 | 1.50 | 0.92 | 0.10 | 1.83 | -0.05 | 0.15 |
| 115 | 0.07 | 0.36 | 0.12 | -0.01 | -0.56 | -0.47 | -0.57 | -0.77 | -1.31 | 2.42 |
| 116 | 0.36 | 0.65 | 1.28 | 2.81 | 2.92 | 1.19 | 1.97 | 3.39 | 3.79 | 0.94 |
| 117 | 0.33 | 0.84 | 0.64 | 2.28 | 3.62 | 0.66 | 1.34 | 2.90 | 3.61 | -0.78 |
| 118 | 0.29 | 1.20 | 1.11 | 3.16 | 3.60 | 0.79 | 1.45 | 2.40 | 2.57 | 5.59 |
| 119 | 0.06 | 0.27 | 0.27 | 0.47 | 2.00 | -0.24 | 0.16 | 0.72 | 1.84 | 0.51 |
| 120 | 0.18 | 0.25 | 0.21 | -1.50 | -1.58 | -0.69 | -0.98 | -2.10 | -2.36 | -0.24 |
| 121 | 0.36 | -0.02 | 0.34 | 2.05 | 2.85 | -0.26 | 0.06 | 0.17 | 0.81 | 0.57 |
| 122 | 0.36 | 0.05 | 0.64 | -0.51 | 1.25 | -0.02 | -0.04 | -0.09 | -0.21 | 2.24 |
| 123 | -0.43 | -0.34 | -0.15 | -1.41 | -1.69 | -1.15 | -1.47 | -2.94 | -3.03 | -2.67 |
| 124 | -0.60 | -0.15 | -0.14 | 0.90 | 0.64 | -0.60 | -0.27 | -0.43 | 0.21 | -1.83 |
| 125 | 0.23 | 0.02 | 0.01 | -0.39 | -1.80 | -0.07 | -0.36 | -0.92 | -1.50 | -3.54 |
| 126 | 0.02 | -0.75 | -0.67 | -2.67 | -4.55 | -1.09 | -1.37 | -2.01 | -4.26 | -4.53 |
| 127 | 0.79 | 1.07 | 1.47 | 3.20 | 3.90 | 1.05 | 1.81 | 2.35 | 2.71 | 5.67 |
| 128 | 0.05 | 0.30 | -0.74 | 0.08 | -0.01 | 0.77 | 1.05 | 0.38 | 0.52 | 3.74 |
| 129 | 0.32 | 0.37 | 0.38 | 0.17 | -0.09 | 0.43 | 0.51 | -0.03 | -0.94 | 4.21 |
| 130 | 0.44 | 0.39 | -0.04 | -1.37 | -2.78 | -0.44 | -1.17 | -1.88 | -3.55 | -3.36 |
| 131 | 0.49 | 0.48 | 0.63 | -0.44 | -0.18 | 0.42 | 0.71 | 0.85 | 1.08 | -2.48 |
| 132 | 0.50 | 0.26 | 0.35 | -1.21 | -1.61 | -0.03 | -0.45 | -1.81 | -2.40 | -4.95 |

Trait abbreviations are as illustrated in S1 Table. For QTL analyses, Box-Cox transformation {[(μ + *x*)^λ^ – 1] / λ} was applied to traits *H*_10_, *H*_23_, *H*_32_, *H*_44_, *H*_56_, *D*_23_, and *D*_32_ with λ values being 3.5, 7.0, 6.8, 3.0, 2.9, 3.4, and 2.7, respectively, where μ is the trait mean and *x* is the BLUP value. For the rest traits *D*_44_, *D*_56_, and *WD*_56_, a trait value was adjusted as sum of the BLUP value and the trait mean.

^a^ Mean of the trait.
