# Supplementary material for: Comparative Genomics Analyses Reveal Extensive Chromosome Colinearity and Novel Quantitative Trait Loci in Eucalyptus
Source: PLoS One. 2015 Dec 22;10(12):e0145144. doi: 10.1371/journal.pone.0145144 (PMC4687840; doi:10.1371/journal.pone.0145144)
Supplement: S10 Table — (DOC) [file pone.0145144.s012.doc]

**S10 Table. Numbers of common, non-syntenic, and non-colinear markers in *E. urophylla* as compared with prior DArT-based genetic maps of *Eucalyptus*, including *E. grandis* × *E. urophylla* F_1_ Full map (GU1) [13], *E. grandis* × *E. urophylla* pseudo-backcross F_2_ consensus map (GU2) [12], and *E. globulus* Lighthouse F_2_ map (Glob**) [10].

| **LG** | **Common markers** | | | |  | **Non-syntenic markers** | | |  | **Non-colinear markers** | | |
| --- | --- | --- | --- | --- | --- | --- | --- | --- | --- | --- | --- | --- |
|  | **GU1** | **GU2** | **Glob** | **GU1/GU2/Glob** |  | **GU1** | **GU2** | **Glob** |  | **GU1** | **GU2** | **Glob** |
| 1 | 20 | 18 | 12 | 36 |  | 0 | 0 | 0 |  | 8 | 1 | 0 |
| 2 | 26 | 32 | 17 | 48 |  | 0 | 0 | 0 |  | 6 | 6 | 1 |
| 3 | 38 | 43 | 15 | 62 |  | 1 | 3 | 1 |  | 10 | 10 | 3 |
| 4 | 5 | 12 | 17 | 21 |  | 0 | 0 | 1 |  | 1 | 1 | 0 |
| 5 | 29 | 27 | 12 | 39 |  | 2 | 2 | 1 |  | 5 | 5 | 1 |
| 6 | 23 | 18 | 7 | 40 |  | 1 | 1 | 1 |  | 4 | 1 | 0 |
| 7 | 32 | 18 | 21 | 42 |  | 0 | 0 | 0 |  | 12 | 3 | 5 |
| 8 | 48 | 34 | 12 | 65 |  | 0 | 1 | 3 |  | 16 | 1 | 0 |
| 9 | 15 | 24 | 14 | 27 |  | 0 | 0 | 0 |  | 6 | 4 | 2 |
| 10 | 26 | 17 | 6 | 33 |  | 0 | 1 | 1 |  | 7 | 1 | 0 |
| 11 | 23 | 21 | 7 | 30 |  | 0 | 0 | 0 |  | 1 | 4 | 0 |
| Total (%) | 285 | 264 | 140 | 443 |  | 4 (1.4) | 8 (3.0) | 8 (5.7) |  | 76 (27.0^a^) | 37 (14.3^a^) | 12 (9.1^a^) |

References could be found in the text. Within subgenus *Symphyomyrtus*, *E. urophylla* and *E. grandis* belong to section *Latoangulatae*, and *E. globulus* belongs to section *Latoangulatae*.

^a^ The percentage of syntenic markers.
