# Supplementary material for: Comparative Genomics Analyses Reveal Extensive Chromosome Colinearity and Novel Quantitative Trait Loci in Eucalyptus
Source: PLoS One. 2015 Dec 22;10(12):e0145144. doi: 10.1371/journal.pone.0145144 (PMC4687840; doi:10.1371/journal.pone.0145144)
Supplement: S11 Table — (DOC) [file pone.0145144.s013.doc]

**S11 Table. Numbers of common, non-syntenic, and non-colinear markers in *E. tereticornis* as compared with prior DArT-based genetic maps of *Eucalyptus*, including *E. grandis* × *E. urophylla* F_1_ Full map (GU1) [13], *E. grandis* × *E. urophylla* pseudo-backcross F_2_ consensus map (GU2) [12], and *E. globulus* Lighthouse F_2_ map (Glob**) [10].

| **LG** | **Common markers** | | | |  | **Non-syntenic markers** | | |  | **Non-colinear markers** | | |
| --- | --- | --- | --- | --- | --- | --- | --- | --- | --- | --- | --- | --- |
|  | **GU1** | **GU2** | **Glob** | **GU1/GU2/Glob** |  | **GU1** | **GU2** | **Glob** |  | **GU1** | **GU2** | **Glob** |
| 1 | 13 | 12 | 0 | 17 |  | 1 | 1 | 0 |  | 3 | 0 | 0 |
| 2 | 18 | 17 | 9 | 26 |  | 0 | 2 | 0 |  | 4 | 3 | 0 |
| 3 | 38 | 42 | 12 | 56 |  | 0 | 1 | 0 |  | 3 | 6 | 0 |
| 4 | 7 | 19 | 2 | 24 |  | 0 | 0 | 1 |  | 3 | 2 | 0 |
| 5 | 23 | 31 | 18 | 38 |  | 1 | 1 | 3 |  | 4 | 3 | 0 |
| 6 | 10 | 7 | 3 | 14 |  | 1 | 0 | 0 |  | 0 | 0 | 0 |
| 7 | 26 | 19 | 12 | 38 |  | 3 | 4 | 0 |  | 7 | 0 | 3 |
| 8 | 21 | 30 | 12 | 36 |  | 0 | 0 | 0 |  | 3 | 3 | 0 |
| 9 | 8 | 6 | 5 | 10 |  | 0 | 0 | 0 |  | 5 | 2 | 0 |
| 10 | 22 | 6 | 7 | 22 |  | 0 | 0 | 1 |  | 6 | 0 | 0 |
| 11 | 26 | 27 | 11 | 39 |  | 0 | 0 | 1 |  | 7 | 3 | 0 |
| Total (%) | 212 | 216 | 91 | 320 |  | 6 (2.8) | 9 (4.2) | 6 (6.6) |  | 45 (21.8^a^) | 22 (10.6^a^) | 3 (3.5^a^) |

References could be found in the text. Within subgenus *Symphyomyrtus*, *E. urophylla* and *E. grandis* belong to section *Latoangulatae*, and *E. globulus* belongs to section *Latoangulatae*.

^a^ The percentage of syntenic markers.
