# Supplementary material for: Comparative Genomics Analyses Reveal Extensive Chromosome Colinearity and Novel Quantitative Trait Loci in Eucalyptus
Source: PLoS One. 2015 Dec 22;10(12):e0145144. doi: 10.1371/journal.pone.0145144 (PMC4687840; doi:10.1371/journal.pone.0145144)
Supplement: S12 Table — (DOC) [file pone.0145144.s014.doc]

**S12 Table. Unique markers in *E. urophylla* (Ur) genetic map as compared with prior SSR- and DArT-based genetic maps of *Eucalyptus*, including *E. grandis* × *E. urophylla* F_1_ Full map (GU1) [13], *E. grandis* × *E. urophylla* pseudo-backcross F_2_ consensus map (GU2) [12], and *E. globulus* Lighthouse F_2_ map (Glob) [10].**

| **LG** | **Ur vs GU1** | | | | |  | **Ur vs GU2** | | | | |  | **Ur vs Glob** | | | | |  | **Ur vs GU1&GU2&Glob** | | | | |
| --- | --- | --- | --- | --- | --- | --- | --- | --- | --- | --- | --- | --- | --- | --- | --- | --- | --- | --- | --- | --- | --- | --- | --- |
|  | **DArT** | **gSSR** | **EST- SSR** | **EST- CAPS** | **Sub-total** |  | **DArT** | **gSSR** | **EST- SSR** | **EST- CAPS** | **Sub-total** |  | **DArT** | **gSSR** | **EST- SSR** | **EST- CAPS** | **Sub-total** |  | **DArT** | **gSSR** | **EST- SSR** | **EST- CAPS** | **Sub- total** |
| 1 | 20 | 5 | 6 | 1 | 32 |  | 22 | 5 | 6 | 1 | 34 |  | 28 | 5 | 6 | 1 | 40 |  | 6 | 3 | 6 | 1 | 16 |
| 2 | 37 | 5 | 7 | 4 | 53 |  | 31 | 5 | 7 | 4 | 47 |  | 44 | 7 | 7 | 4 | 62 |  | 16 | 4 | 7 | 4 | 31 |
| 3 | 46 | 1 | 13 | 2 | 62 |  | 39 | 3 | 13 | 2 | 57 |  | 66 | 4 | 13 | 2 | 85 |  | 22 | 1 | 13 | 2 | 38 |
| 4 | 24 | 1 | 4 | 1 | 30 |  | 15 | 3 | 4 | 1 | 23 |  | 19 | 4 | 4 | 1 | 28 |  | 8 | 1 | 4 | 1 | 14 |
| 5 | 22 | 0 | 5 | 0 | 27 |  | 22 | 2 | 5 | 0 | 29 |  | 38 | 1 | 5 | 0 | 44 |  | 12 | 0 | 5 | 0 | 17 |
| 6 | 31 | 2 | 16 | 10 | 59 |  | 35 | 3 | 16 | 10 | 64 |  | 46 | 3 | 16 | 10 | 75 |  | 14 | 2 | 16 | 10 | 42 |
| 7 | 16 | 2 | 4 | 2 | 24 |  | 29 | 3 | 4 | 2 | 38 |  | 26 | 3 | 4 | 2 | 35 |  | 6 | 2 | 4 | 2 | 14 |
| 8 | 31 | 4 | 12 | 4 | 51 |  | 46 | 3 | 12 | 4 | 65 |  | 65 | 6 | 12 | 4 | 87 |  | 16 | 2 | 12 | 4 | 34 |
| 9 | 16 | 1 | 7 | 5 | 29 |  | 7 | 1 | 7 | 5 | 20 |  | 16 | 2 | 7 | 5 | 30 |  | 4 | 1 | 7 | 5 | 17 |
| 10 | 15 | 1 | 8 | 3 | 27 |  | 22 | 3 | 8 | 3 | 36 |  | 32 | 4 | 8 | 3 | 47 |  | 9 | 0 | 8 | 3 | 20 |
| 11 | 8 | 4 | 8 | 1 | 21 |  | 10 | 4 | 8 | 1 | 23 |  | 23 | 5 | 8 | 1 | 37 |  | 2 | 3 | 8 | 1 | 14 |
| Sub- total | 266 | 26 | 90 | 33 |  |  | 278 | 35 | 90 | 33 |  |  | 403 | 44 | 90 | 33 |  |  | 115 | 19 | 90 | 33 |  |
| Total |  |  |  |  | 415 |  |  |  |  |  | 436 |  |  |  |  |  | 570 |  |  |  |  |  | 257 |

References could be found in the text.
