# Supplementary material for: Comparative Genomics Analyses Reveal Extensive Chromosome Colinearity and Novel Quantitative Trait Loci in Eucalyptus
Source: PLoS One. 2015 Dec 22;10(12):e0145144. doi: 10.1371/journal.pone.0145144 (PMC4687840; doi:10.1371/journal.pone.0145144)
Supplement: S13 Table — (DOC) [file pone.0145144.s015.doc]

**S13 Table. Unique markers in *E. tereticornis* (Te) genetic map as compared with prior SSR- and DArT-based genetic maps of *Eucalyptus*, including *E. grandis* × *E. urophylla* F_1_ Full map (GU1) [13], *E. grandis* × *E. urophylla* pseudo-backcross F_2_ consensus map (GU2) [12], and *E. globulus* Lighthouse F_2_ map (Glob) [10].**

| **LG** | **Te vs GU1** | | | | |  | **Te vs GU2** | | | | |  | **Te vs Glob** | | | | |  | **Te vs GU1&GU2&Glob** | | | | |
| --- | --- | --- | --- | --- | --- | --- | --- | --- | --- | --- | --- | --- | --- | --- | --- | --- | --- | --- | --- | --- | --- | --- | --- |
|  | **DArT** | **gSSR** | **EST- SSR** | **EST- CAPS** | **Sub-total** |  | **DArT** | **gSSR** | **EST- SSR** | **EST- CAPS** | **Sub-total** |  | **DArT** | **gSSR** | **EST- SSR** | **EST- CAPS** | **Sub-total** |  | **DArT** | **gSSR** | **EST- SSR** | **EST- CAPS** | **Sub- total** |
| 1 | 13 | 4 | 6 | 2 | 25 |  | 12 | 6 | 6 | 2 | 26 |  | 24 | 6 | 6 | 2 | 38 |  | 9 | 4 | 6 | 2 | 21 |
| 2 | 15 | 4 | 14 | 3 | 36 |  | 14 | 6 | 14 | 3 | 37 |  | 24 | 4 | 14 | 3 | 45 |  | 7 | 4 | 14 | 3 | 28 |
| 3 | 30 | 2 | 10 | 2 | 44 |  | 26 | 2 | 10 | 2 | 40 |  | 56 | 1 | 10 | 2 | 69 |  | 14 | 0 | 10 | 2 | 26 |
| 4 | 32 | 0 | 1 | 1 | 34 |  | 19 | 1 | 1 | 1 | 22 |  | 36 | 1 | 1 | 1 | 39 |  | 15 | 0 | 1 | 1 | 17 |
| 5 | 31 | 2 | 6 | 0 | 39 |  | 21 | 4 | 6 | 0 | 31 |  | 34 | 4 | 6 | 0 | 44 |  | 16 | 2 | 6 | 0 | 24 |
| 6 | 8 | 2 | 9 | 5 | 24 |  | 10 | 3 | 9 | 5 | 27 |  | 15 | 2 | 9 | 5 | 31 |  | 5 | 1 | 9 | 5 | 20 |
| 7 | 22 | 1 | 7 | 1 | 31 |  | 29 | 1 | 7 | 1 | 38 |  | 36 | 1 | 7 | 1 | 45 |  | 10 | 1 | 7 | 1 | 19 |
| 8 | 34 | 2 | 16 | 5 | 57 |  | 23 | 4 | 16 | 5 | 48 |  | 40 | 5 | 16 | 5 | 66 |  | 19 | 2 | 16 | 5 | 42 |
| 9 | 14 | 0 | 6 | 1 | 21 |  | 15 | 1 | 6 | 1 | 23 |  | 15 | 2 | 6 | 1 | 24 |  | 12 | 0 | 6 | 1 | 19 |
| 10 | 9 | 1 | 13 | 1 | 24 |  | 23 | 3 | 13 | 1 | 40 |  | 23 | 2 | 13 | 1 | 39 |  | 9 | 1 | 13 | 1 | 24 |
| 11 | 24 | 3 | 8 | 3 | 38 |  | 22 | 4 | 8 | 3 | 37 |  | 38 | 4 | 8 | 3 | 53 |  | 11 | 3 | 8 | 3 | 25 |
| Sub- total | 232 | 21 | 96 | 24 |  |  | 214 | 35 | 96 | 24 |  |  | 341 | 32 | 96 | 24 |  |  | 127 | 18 | 96 | 24 |  |
| Total |  |  |  |  | 373 |  |  |  |  |  | 369 |  |  |  |  |  | 493 |  |  |  |  |  | 265 |

References could be found in the text.
