# Supplementary material for: Comparative Genomics Analyses Reveal Extensive Chromosome Colinearity and Novel Quantitative Trait Loci in Eucalyptus
Source: PLoS One. 2015 Dec 22;10(12):e0145144. doi: 10.1371/journal.pone.0145144 (PMC4687840; doi:10.1371/journal.pone.0145144)
Supplement: S14 Table — (DOC) [file pone.0145144.s016.doc]

**S14 Table. Statistics for alignment of *E. urophylla* and *E. tereticornis* genetic maps with *E. grandis*** genome sequence V1.1.

| **LG** | ***E. urophylla*** | | | | | | | |  | ***E. tereticornis*** | | | | | | | |
| --- | --- | --- | --- | --- | --- | --- | --- | --- | --- | --- | --- | --- | --- | --- | --- | --- | --- |
|  | **Coverage** | | **kb.cM^-1^** | **No. NAS** | **No. no hit** | **No. aligned** | **No. non- syntenic^a^** | **No. non- colinear** |  | **Coverage** | | **kb.cM^-1^** | **No. NAS** | **No. no hit** | **No. aligned** | **No. non- syntenic^a^** | **No. non- colinear** |
|  | **Mb** | **%** |  |  |  |  |  |  |  | **Mb** | **%** |  |  |  |  |  |  |
| 1 | 40.23 | 99.8 | 580.5 | 4 | 1 | 47 | 2 | 17 |  | 39.17 | 97.2 | 373.4 | 0 | 0 | 38 | 2 | 7 |
| 2 | 62.59 | 97.4 | 478.5 | 7 | 1 | 71 | 1 | 24 |  | 63.26 | 98.5 | 405.5 | 4 | 0 | 50 | 4^b^ | 8 |
| 3 | 79.81 | 99.6 | 784.0 | 9 | 0 | 91 | 5 | 32 |  | 79.81 | 99.6 | 750.1 | 5 | 0 | 77 | 1 | 14 |
| 4 | 40.08 | 95.5 | 489.4 | 3 | 0 | 32 | 1^b^ | 4 |  | 40.64 | 96.8 | 538.3 | 1 | 1 | 39 | 0 | 4 |
| 5 | 74.07 | 99.1 | 730.5 | 3 | 0 | 53 | 8 | 9 |  | 73.12 | 97.9 | 696.4 | 7 | 0 | 55 | 5 | 9 |
| 6 | 53.26 | 98.8 | 398.7 | 1 | 0 | 81 | 4 | 15 |  | 52.95 | 98.3 | 480.1 | 1 | 0 | 33 | 0 | 8 |
| 7 | 52.14 | 99.4 | 465.1 | 5 | 0 | 51 | 2 | 17 |  | 51.90 | 99.0 | 507.8 | 5 | 2 | 50 | 5 | 7 |
| 8 | 68.17 | 91.7 | 406.5 | 14 | 0 | 85 | 5^b^ | 25 |  | 73.86 | 99.4 | 438.6 | 5 | 1 | 72 | 4^c^ | 15 |
| 9 | 38.40 | 98.4 | 395.5 | 7 | 0 | 37 | 1 | 9 |  | 38.40 | 98.4 | 337.1 | 3 | 1 | 25 | 2 | 5 |
| 10 | 36.16 | 91.9 | 372.0 | 5 | 0 | 48 | 1 | 10 |  | 38.23 | 97.1 | 388.9 | 1 | 0 | 45 | 0 | 3 |
| 11 | 43.63 | 95.9 | 378.4 | 0 | 1 | 43 | 2 | 11 |  | 42.33 | 93.0 | 421.2 | 2 | 2 | 60 | 1 | 18 |
| Total (%) | 588.54 | 97.1 | 487.1 | 58 (8.3) | 3 (0.5^d^) | 639 (99.5^d^) | 32 (5.0^e^) | 173 (28.5^f^) |  | 593.67 | 97.7 | 478.2 | 34 (5.8) | 7 (1.3^d^) | 544 (98.7^d^) | 24 (4.4^e^) | 98 (18.8^f^) |

LG, linkage group; NAS, not available sequence.

^a^ The markers with no hit were excluded.

^b^ One marker aligned to a small scaffold was included.

^c^ Three markers aligned to small scaffolds were included.

^d^ The percentage of mapped markers available for sequence.

^e^ The percentage of aligned markers.

^f^ The percentage of syntenic markers.
