# Supplementary material for: Comparative Genomics Analyses Reveal Extensive Chromosome Colinearity and Novel Quantitative Trait Loci in Eucalyptus
Source: PLoS One. 2015 Dec 22;10(12):e0145144. doi: 10.1371/journal.pone.0145144 (PMC4687840; doi:10.1371/journal.pone.0145144)
Supplement: S15 Table — (DOC) [file pone.0145144.s017.doc]

**S15 Table. Comparison of QTLs detected on the homologous linkage groups across SSR- and DArT-based studies in *Eucalyptus***.

| **Trait** | **LG** | **This study** | | | |  | **Kullan et al. [17]** | | |  | **Freeman et al. [18]** | | |
| --- | --- | --- | --- | --- | --- | --- | --- | --- | --- | --- | --- | --- | --- |
|  |  | **Ur / Te** | **Age (yr)^a^** | **GMP (cM)** | **APP (Mb)** |  | **Age (yr)** | **GMP (cM)** | **APP (Mb)** |  | **Age (yr)** | **GMP (cM)** | **APP (Mb)** |
| *D* | 2 | Ur | 4.7 | 48.5 | 18.4 |  | - | - | - |  | 7.0 | 51.0 | 36.9 |
|  | 2 | Te | 4.7 | 120.7 | 59.6 |  | - | - | - |  | - | - | - |
|  | 5 | Te | 4.7 | 25.5 | 7.8 |  | - | - | - |  | 7.0 | 47.8 | 12.0 |
|  | 5 | Ur | 4.7 | 55.6 | 51.5 |  | - | - | - |  | - | - | - |
|  | 8 | Ur | 4.7 | 5.4 | 4.1 |  | - | - | - |  | 7.0 | 55.9 | 42.7 |
|  | 8 | - | - | - | - |  | - | - | - |  | 7.0 | 80.7 | 57.2 |
| *WD* | 2 | Te | 4.7 | 120.8 | 59.6 |  | 4.0 | 99.2 | 63.6 |  | 7.0 | 33.5 | 45.8 |
|  | 2 | - | - | - | - |  | - | - | - |  | 7.0 | 57.5 | 45.1 |
|  | 2 | - | - | - | - |  | - | - | - |  | 7.0 | 119.6 | 62.3 |
|  | 4 | Ur | 4.7 | 7.9 | 4.5 |  | 4.0 | 12.5 | 11.6 |  | - | - | - |
|  | 4 | - | - | - | - |  | 4.0 | 39.7 | 31.5 |  | - | - | - |
|  | 5 | Te | 4.7 | 62.4 | 46.8 |  | - | - | - |  | 7.0 | 33.3 | 12.3 |
|  | 6 | Ur | 4.7 | 64.1 | 34.5 |  | 4.0 | 20.1 | 10.0 |  | 7.0 | 72.8 | 29.5 |
|  | 6 | - | - | - | - |  | - | - | - |  | 7.0 | 97.5 | 41.1 |
|  | 8 | Ur | 4.7 | 49.2 | 13.0 |  | 4.0 | 32.0 | 17.8 |  | 7.0 | 37.5 | 18.9 |
|  | 8 | - | - | - | - |  | 4.0 | 75.7 | 59.0 |  | 7.0 | 64.3 | 47.7 |
|  | 8 | - | - | - | - |  | - | - | - |  | 7.0 | 93.8 | 62.6 |
|  | 9 | Ur | 4.7 | 93.9 | 38.2 |  | 4.0 | 41.5 | 25.8 |  | 7.0 | 20.4 | 29.9 |
|  | 9 | - | - | - | - |  | 4.0 | 65.5 | 34.2 |  | - | - | - |

*D*, diameter at 1.3 m height; *WD*, basic wood density; GMP, genetic map position; APP, approximate physical position. The hyphen '-' indicates an inapplicable case. References could be found in the text.

^a^ The age of 4.7 is equal to 56 months.
